# Core *N*-Glycan Structures Are Critical for the Pathogenicity of Cryptococcus neoformans by Modulating Host Cell Death

**DOI:** 10.1128/mBio.00711-20

**Published:** 2020-05-12

**Authors:** Eun Jung Thak, Su-Bin Lee, Shengjie Xu-Vanpala, Dong-Jik Lee, Seung-Yeon Chung, Yong-Sun Bahn, Doo-Byoung Oh, Mari L. Shinohara, Hyun Ah Kang

**Affiliations:** a Department of Life Science, Chung-Ang University, Seoul, South Korea; b Department of Biotechnology, College of Life Science and Biotechnology, Yonsei University, Seoul, South Korea; c Korea Research Institute of Bioscience and Biotechnology, Daejeon, South Korea; d Department of Immunology, Duke University School of Medicine, Durham, North Carolina, USA; e Department of Microbiology and Molecular Genetics, Duke University School of Medicine, Durham, North Carolina, USA; Duke University Medical Center

**Keywords:** *Cryptococcus neoformans*, *N*-linked protein glycosylation, *ALG*, fungal pathogenesis

## Abstract

Cryptococcus neoformans is a human-pathogenic fungal pathogen that causes life-threatening meningoencephalitis in immunocompromised individuals. To investigate the roles of *N*-glycan core structure in cryptococcal pathogenicity, we constructed mutant strains of C. neoformans with defects in the assembly of lipid-linked *N*-glycans in the luminal side of the endoplasmic reticulum (ER). Deletion of *ALG3* (*alg3*Δ), which encodes dolichyl-phosphate-mannose (Dol-P-Man)-dependent α-1,3-mannosyltransferase, resulted in the production of truncated neutral *N*-glycans carrying five mannose residues as a major species. Despite moderate or nondetectable defects in virulence-associated phenotypes *in vitro*, the *alg3*Δ mutant was avirulent in a mouse model of systemic cryptococcosis. Notably, the mutant did not show defects in early stages of host cell interaction during infection, including attachment to lung epithelial cells, opsonic/nonopsonic phagocytosis, and manipulation of phagosome acidification. However, the ability to drive macrophage cell death was greatly decreased in this mutant, without loss of cell wall remodeling capacity. Furthermore, deletion of *ALG9* and *ALG12*, encoding Dol-P-Man-dependent α-1,2-mannosyltransferases and α-1,6-mannosyltransferases, generating truncated core *N*-glycans with six and seven mannose residues, respectively, also displayed remarkably reduced macrophage cell death and *in vivo* virulence. However, secretion levels of interleukin-1β (IL-1β) were not reduced in the bone marrow-derived dendritic cells obtained from *Asc*- and *Gsdmd*-deficient mice infected with the *alg3*Δ mutant strain, excluding the possibility that pyroptosis is a main host cell death pathway dependent on intact core *N*-glycans. Our results demonstrated *N*-glycan structures as a critical feature in modulating death of host cells, which is exploited by as a strategy for host cell escape for dissemination of C. neoformans.

## Abstract (precis)

We previously reported that the outer mannose chains of *N*-glycans are dispensable for the virulence of C. neoformans, which is in stark contrast to findings for the other human-pathogenic yeast, Candida albicans. Here, we present evidence that an intact core *N*-glycan structure is required for C. neoformans pathogenicity by systematically analyzing *alg3Δ, alg9Δ*, and *alg12Δ* strains that have defects in lipid-linked *N-*glycan assembly and in *in vivo* virulence. The *alg* null mutants producing truncated core *N*-glycans were defective in inducing host cell death after phagocytosis, which is triggered as a mechanism of pulmonary escape and dissemination of C. neoformans, thus becoming inactive in causing fatal infection. The results clearly demonstrated the critical features of the *N*-glycan structure in mediating the interaction with host cells during fungal infection. The delineation of the roles of protein glycosylation in fungal pathogenesis not only provides insight into the glycan-based fungal infection mechanism but also will aid in the development of novel antifungal agents.

## INTRODUCTION

Glycosylation is the most universal and diverse posttranslational modification that affects protein activity, folding, stability, transport, and immunogenicity (1, 2). Particularly, glycans assembled on cell surface glycoproteins of fungal pathogens modulate the efficiency of pathogen adhesion to and interaction with host cells during infection (3–7). *N*-linked glycans on yeast glycoproteins are mostly high-mannose types with exclusive addition of mannose residues and are different from the mammalian hybrid-type and complex-type *N*-glycans that contain other sugars besides mannose (8). Although the core structures of *N-*glycans assembled in the endoplasmic reticulum (ER) are highly conserved among eukaryotes, *N*-linked glycosylation in the Golgi apparatus is differentially processed even among closely related fungal species (9). *O*-Glycans of Saccharomyces cerevisiae consist of short oligosaccharides that mostly contain one to four mannose residues. The common core structure of *O*-glycans, an α1,2-linked mannotriose, is differentially expanded in different fungal species (10). Recently, the critical role of protein glycosylation in cell wall integrity, morphogenesis, virulence, and immune recognition has been reported in medically important fungal pathogens, including several *Candida* species, such as Candida albicans (3, 5, 11, 12), Candida glabrata (13), and Candida parapsilosis (14); the dimorphic fungus Histoplasma capsulatum (15); and the filamentous fungus Aspergillus fumigatus (16).

Cryptococcus neoformans is a basidiomycetous human-pathogenic fungal pathogen that causes fatal infection of the central nervous system in immunocompromised patients. The World Health Organization reported that cryptococcal meningoencephalitis causes 15% of AIDS-related deaths globally (17). C. neoformans infection initiates with the inhalation of spores and desiccated yeast cells, which first encounter resident alveolar macrophages in the lungs (18). Macrophages play critical roles in host defense by damaging or killing pathogens and activating host immune responses (19, 20). In general, following the engulfment of microbes by macrophages, phagosomes undergo maturation by acidification through fusion with lysosomes, ultimately leading to the degradation of infectious microbes. However, C. neoformans has evolved to avoid intracellular killing, replicate within host phagocytes, and escape from phagocytes either via host cell lysis (lytic exocytosis) or via a nonlytic process (vomocytosis) (21). Modulation of phagolysosomal membrane permeabilization (PMP) is a critical event in determining the outcome of the C. neoformans-macrophage interaction. Induced PMP enhances macrophages to trigger lytic exocytosis, while nonlytic exocytosis is common in those without PMP (22). Phagocyte escape is crucial for dissemination of C. neoformans cells to other host tissues, particularly in the central nervous system, and for disease progression (23). *Cryptococcus* possesses several virulence factors that support immune evasion and enhance its ability to thrive within phagocytes, including the pigment melanin, extracellular enzymes such as phospholipase and urease (24–26), and the ability to grow at the mammalian host temperature (27). In particular, its polysaccharide capsule inhibits phagocytosis (28). Mannoproteins secreted or localized on the cell surface, such as Cig1 (29) and MP84 (7), are also closely associated with the pathogenicity of C. neoformans.

We previously reported the unique structure and biosynthetic pathway of C. neoformans *N*-glycans assembled on mannoproteins (30). C. neoformans has serotype-specific high-mannose-type *N*-glycans with or without a β-1,2-xylose residue, which is attached to the trimannosyl core of *N*-glycans. Interestingly, the neutral *N*-glycans of serotype A (strain H99) and serotype D (strain JEC21) contain a xylose residue, whereas those of serotype B (strain R265) are substantially shorter and have no xylose residue. Moreover, the acidic *N*-glycans of C. neoformans contain xylose phosphates attached to mannose residues in the *N*-glycan core and outer chain, whereas most yeast species contain phosphomannan as acidic sugar (30). In C. albicans and C. parapsilosis, loss of the *N*-glycan outer chain by deletion of *OCH1* (*och1*Δ), which encodes a key enzyme initiating the α1,6-linked polymannose backbone of *N*-glycans in the Golgi apparatus, attenuates virulence (3, 14). Moreover, in C. albicans, even subtle alteration of mannose outer chains of *N*-glycans, generated by deletion of genes involved in the extension of *N*-glycan outer chains, such as the *MNN2* family, *MNN5*, and *MNN10* of the *MNN1* family, results in severe attenuation of virulence (4, 5, 11, 12). In the case of C. glabrata, deletion of the genes involved in the extension of outer chains of *N*-glycans differentially affects virulence (13). In contrast, in C. neoformans, the *och1*Δ deletion affects virulence only marginally (30). Those studies suggested that the contribution of the outer chain of *N*-glycans to fungal pathogenicity is species dependent.

In this study, we assessed the importance of the core *N-*glycan structure in the virulence of C. neoformans. In eukaryotic cells, the core *N-*glycan is assembled in a highly defined way by a series of specific glycosyltransferases localized at the ER and encoded by the asparagine-linked glycosylation (*ALG*) pathway genes (31). Therefore, we deleted C. neoformans *ALG3*, *ALG9*, and *ALG12*, which encode dolichyl-phosphate-mannose (Dol-P-Man)-dependent mannosyltransferases involved in lipid-linked *N*-glycan biosynthesis in the ER lumen, which results in the production of a truncated *N*-glycan core. Using the *alg* null mutants, we systematically evaluated the effects of altered core *N*-glycan structures on C. neoformans pathogenicity by assessing virulence-associated phenotypes *in vitro* and in a mouse model of systemic cryptococcosis as well as the effects on host immune cell interactions. Here, we present data supporting the idea of a critical role of the core *N*-glycan structure in inducing macrophage cell death after phagocytosis, which might be triggered as a host cell escape mechanism, in the late stage of lung infection.

## RESULTS

### *ALG3* deletion causes a defect in the first step of lipid-linked oligosaccharide biosynthesis in the ER lumen of C. neoformans.

**FIG 1:**
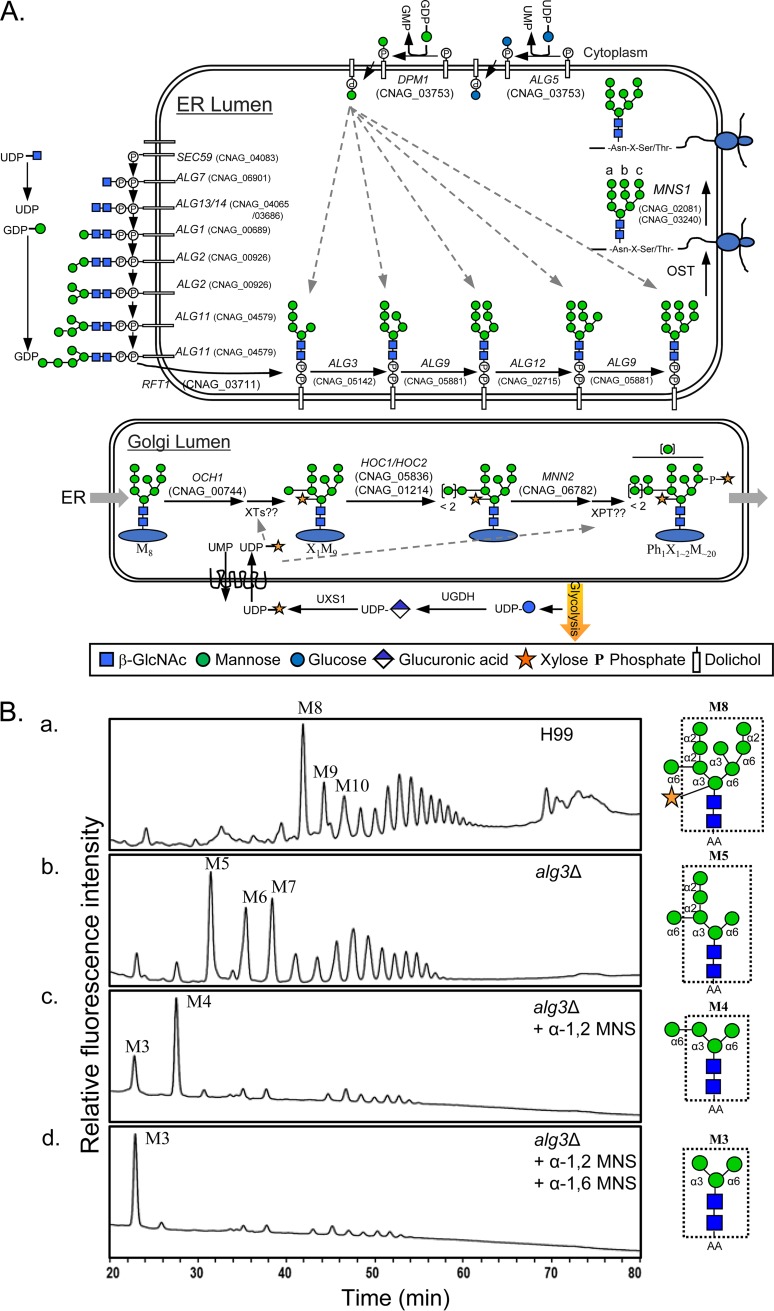
C. neoformans Alg3 is involved in lipid-linked *N*-glycan biosynthesis within the ER lumen. (A) Schematic representation of the *N*-glycosylation pathway in C. neoformans. The C. neoformans genes involved in *N*-glycosylation were identified by BLAST analysis of the genome database of C. neoformans serotype A (H99) with S. cerevisiae genes participating in *N*-glycan biosynthesis. After the final precursor of Dol-PP-glycan, Dol-PP-GlcNAc_2_Man_9,_ is transferred to Asn-X-Ser/Thr during protein synthesis, ER α-mannosidase I (Mns1p) removes the terminal α-1,2 mannose residue from the b branch of GlcNAc_2_Man_9_ to yield GlcNAc_2_Man_8_. The C. neoformans genes coding for xylosyltransferase (XT) and xylosylphosphotransferase (XPT), assigned to *N*-glycan modification in the Golgi complex, have not yet been identified. (B) HPLC profiles of neutral *N*-glycans of cell wall mannoproteins (cwMPs) from the WT (panel a) and *alg3*Δ mutant strains (panels b, c, and d). Normal-phase HPLC was conducted using a Shodex Asahipak NH2P-50 4E column (Showa Denko) (0.46 by 25 cm) with solvent A (2% glacial acetic acid–1% tetrahydrofuran–acetonitrile) and solvent B (5% glacial acetic acid–3% triethylamine–1% tetrahydrofuran–HPLC water) at a flow rate of 1.0 ml/min. After sample injection, the proportion of solvent B was maintained at 30% for 5 min and then increased linearly to 70% over 80 min. *N*-Glycans of *alg3*Δ (panel b) were treated serially with α-1,2 mannosidase (panel c) and α-1,6 mannosidase (panel d). The representative structure of the major *N*-glycan species for each sample is presented with indication of the core *N*-glycans in a dotted box.

*N*-Glycan biosynthesis starts with the transfer of *N*-acetylglucosamine phosphate (GlcNAc-P) to Dol-P, followed by the addition of GlcNAc and five mannose (Man) residues, yielding Dol-pyrophosphate (PP)-GlcNAc_2_Man_5_, on the cytoplasmic side of the ER membrane. After translocation of the heptasaccharide moiety into the ER lumen, four more mannoses are added from the lipid-linked donor Dol-P-Man by Alg3, Alg9, and Alg12, generating Dol-PP-GlcNAc_2_Man_9_ (Fig. 1A). To investigate the role of the core *N*-glycan structure in the pathogenicity of C. neoformans, we initially constructed a null mutant with a deletion of *ALG3*, encoding a Dol-P-Man-dependent α-1,3-mannosyltransferase involved in the first step of the assembly of lipid-linked *N*-glycans after translocation of Dol-PP-GlcNAc_2_Man_5_ into the ER lumen (Fig. 1A). The Alg3 protein, encoded by C. neoformans AG_05142 (CNAG_05142), was predicted to have nine transmembrane domains and a KKXX-like motif (ER retention signal) at the C terminus, suggesting that it is localized in the ER membrane (see Fig. S1A in the supplemental material). C. neoformans Alg3 shows 45.5% and 44.5% sequence identity to Aspergillus niger and Schizosaccharomyces pombe Alg3 orthologs, respectively (Fig. S1B). Sequence alignment of various fungal Alg3 orthologs revealed several conserved regions, including the ER retention sequence (Fig. S1C). The *ALG3* gene in C. neoformans strain H99 was deleted by homologous recombination using double-joint PCR (Fig. S2A). In high-performance liquid chromatography (HPLC) profiles of neutral *N*-linked oligosaccharides obtained from cell wall mannoproteins (cwMPs), the M8 (GlcNAc_2_Man_8_) peak, corresponding to *N*-glycans carrying 8 mannose residues, and higher peaks were markedly reduced, whereas the M5 to M7 peaks (GlcNAc_2_Man_5–7_) were increased for the *alg3Δ* mutant compared with that for the wild-type (WT) strain (Fig. 1B, panels a and b). Treatment with α-1,2 mannosidase (MNS) resulted in the convergence of most *N*-glycan peaks at M4 (GlcNAc_2_Man_4_) (Fig. 1B, panel c), and sequential treatment with α-1,2 mannosidase and α-1,6 mannosidase resulted in the shift of the major M4 peak to the M3 (GlcNAc_2_Man_3_) (Fig. 1B, panel d). This suggested that the *alg3*Δ strain is defective in the conversion of Dol-PP-GlcNAc_2_Man_5_ to Dol-PP-GlcNAc_2_Man_6_, which is the first step in attaching a mannose residue to the lipid-linked *N*-oligosaccharide in the ER lumen, generating a truncated *N*-glycan core carrying 5 to 7 mannose residues, among which M5 is extended with additional α-1,2 mannose residues and a single α-1,6-outer-chain mannose residue. The altered *N*-glycan profile of the *alg3Δ* strain was restored to the WT profile after reintroduction of the WT *ALG3* gene (Fig. S2B and C).

10.1128/mBio.00711-20.2 FIG S1 Bioinformatics analysis of Alg3 protein. (A) Domain structure of C. neoformans Alg3. Predicted transmembrane domains and ER retention signals of Alg3 are indicated by slashes and a box, respectively. (B) Sequence identities and similarities between Alg3 homologs of S. cerevisiae (ScAlg3: NP_009471), Hansenula polymorpha (HpAlg3: ABB04524), C. albicans (CaAlg3: XP_712105), A. niger (AnAlg3: XP_001398696), and S. pombe (SpAlg3: NP_593853). (C) Multiple-sequence alignment of Alg3 homologs generated using ClustalW 1.8. Identical residues and conservative amino acid substitutions are indicated by black and gray shading, respectively. The ER retention signal, the KKXX-like (X = any amino acid) motif, is boxed. Download FIG S1, PDF file, 0.1 MB. Copyright © 2020 Thak et al. 2020 Thak et al. This content is distributed under the terms of the Creative Commons Attribution 4.0 International license.

10.1128/mBio.00711-20.3 FIG S2 Disruption of *ALG3* and construction of a complementation strain. (A) Strategy for CNAG_05142 disruption using the nourseothricin acetyltransferase (NAT) split marker. Stable transformants were selected on YPD medium containing nourseothricin (100 μg/ml) and were screened by PCR. (B) Scheme of construction of an *alg3*Δ::*ALG3* complementation strain. (C) HPLC-based neutral glycan profiles of WT, *alg3*Δ, and *alg3*Δ::*ALG3* strains. Download FIG S2, PDF file, 0.2 MB. Copyright © 2020 Thak et al. 2020 Thak et al. This content is distributed under the terms of the Creative Commons Attribution 4.0 International license.

**FIG 2:**
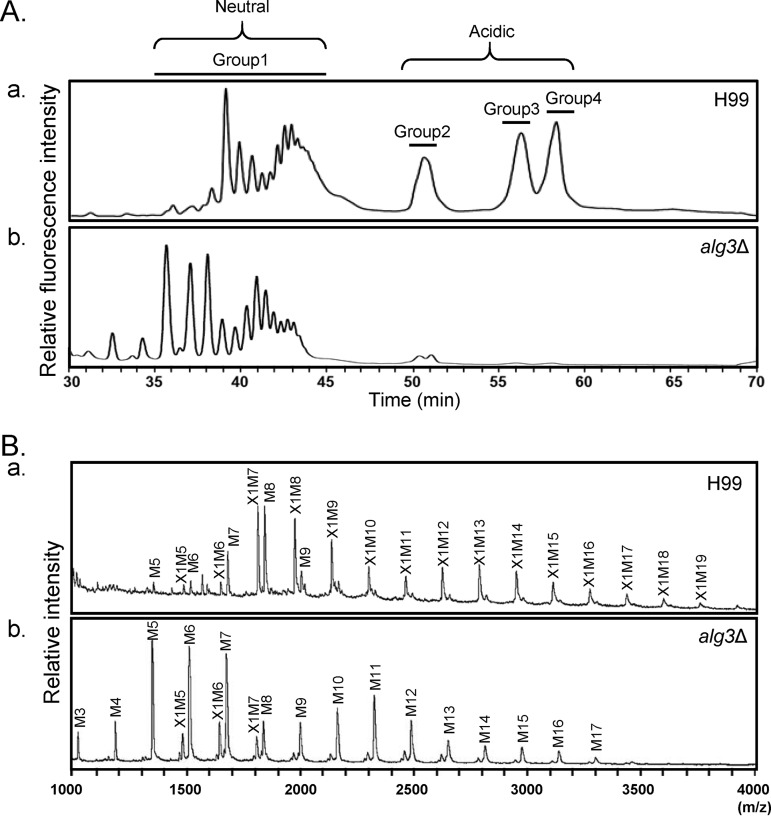
C. neoformans *alg3*Δ produces truncated core *N*-glycans carrying five mannose residues as a major species. (A) HPLC profiles of acidic and neutral *N*-glycans of the WT (panel a) and *alg3*Δ (panel b) strains. For the separation of acidic and neutral *N*-glycans, the proportion of solvent B was maintained at 10% for 5 min and then increased linearly to 90% over 60 min. (B) MALDI-TOF profiles of neutral *N*-glycans. AA-labeled neutral *N*-glycans were selectively collected from group 1 identified on the basis of the HPLC profiles determined as described in the Fig. 2A legend. WT (panel a) and *alg3*Δ mutant (panel b) samples were analyzed by MALDI-TOF spectrometry in the linear negative mode.

HPLC analysis of total *N*-glycans, including neutral and acidic glycans, showed that WT *N*-glycans were separated into one group of neutral glycans (group 1) and three groups of acidic glycans (groups 2, 3, and 4) (Fig. 2A), as previously reported (30). Notably, group 2, 3, and 4 acidic peaks were not detected in the *alg3Δ* mutant, indicating the absence of xylose phosphate addition to *N*-glycans. This was expected because the truncated core *N*-glycans do not possess the sites for xylosylphosphotransferase action (Fig. 1A). For detailed structural analysis of the neutral *N*-glycans, group 1 *N*-glycans were subjected to matrix-assisted laser desorption ionization–time of flight (MALDI-TOF) analysis in the negative mode. Considering that a single xylose residue is attached to the first mannose residue of the trimannosyl core of *N*-glycan, the *alg3Δ* mutant was expected to have a xylose residue attached to the trimannosyl core. Interestingly, however, most of the *N*-glycans of the mutant had no xylose residue (Fig. 2B). These results strongly indicated that xylose residue attachment with the truncated core *N*-glycans might be inefficient. Consequently, truncated neutral *N-*glycans without xylose and xylosylphosphate residues are major forms in the *alg3*Δ mutant.

### *ALG3* deletion affects *N*-glycosylation of the secretory glycoproteins Plb1 and MP98.

**FIG 3:**
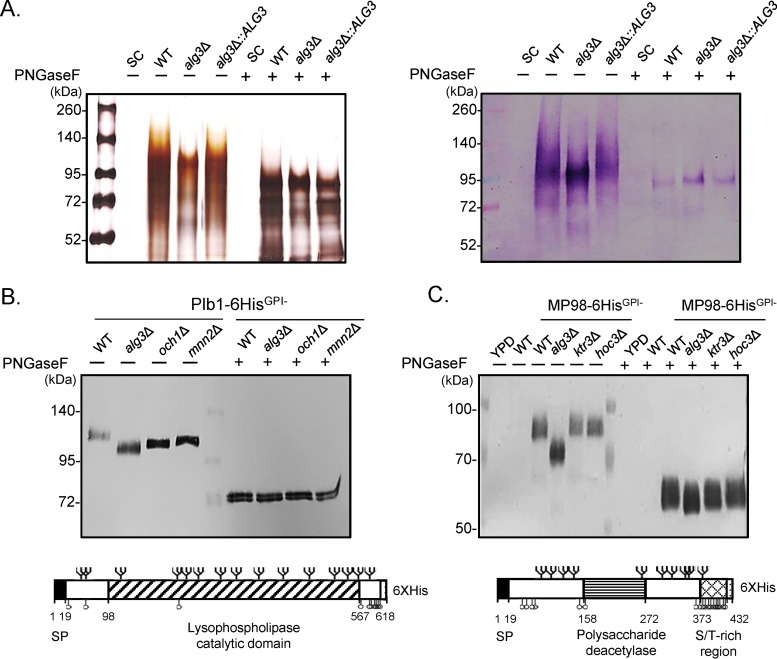
Secreted proteins in C. neoformans *alg3*Δ mutant show truncated *N*-glycosylation patterns. (A) Silver staining and lectin blotting of SDS-polyacrylamide gels containing proteins from culture supernatants of the WT, *alg3*Δ, and *alg3*Δ::*ALG3* strains. Yeast cells were cultivated in SC medium for 24 h and harvested. The culture supernatants were subjected to trichloroacetic acid (TCA) precipitation, silver staining (left), or blotting (right) with Galanthus nivalis agglutinin (Roche), which recognizes terminal α1,2-, α1,3-, and α1,6-linked mannose residues. (B) Western blot of Plb1 proteins secreted from several *N*-glycosylation mutants of C. neoformans, including the *alg3*Δ, *och1*Δ, and *mnn2*Δ strains. (C) Western blot of MP98 secreted from several *N*-/*O*-glycosylation mutants of C. neoformans, including the *alg3*Δ, *ktr3*Δ, and *hoc3*Δ strains. Yeast cells were cultivated in YPD medium for 24 h and harvested, and the culture supernatants were subjected to TCA precipitation and Western blot analysis performed with anti-His antibody (Santa Cruz Biotechnology). SP, signal peptide; branch symbol, putative *N*-glycosylation site; circle, putative *O*-glycosylation site. *N*- or *O*-glycosylation sites were predicted using NetNGlyc 1.0, YinOYang 1.2, and NetOGlyc 4.0.

We analyzed the effect of *ALG3* deletion on protein glycosylation by comparatively analyzing the glycosylation patterns of whole proteins secreted from WT and *alg3Δ* mutant strains by silver staining and lectin blotting of sodium dodecyl sulfate (SDS)-polyacrylamide gels (Fig. 3A). On silver-stained SDS-polyacrylamide gels, the secreted proteome from the WT displayed a broad range of proteins with high molecular weight (MW), whereas the *alg3Δ* mutant proteome showed patterns of significantly faster migration, reflecting the presence of underglycosylated forms of secretory proteins. After treatment with peptide:N-glycosidase F (PNGase F), which removes *N*-linked glycans from glycoproteins, most heterogeneous high-MW protein bands were converted to homogenous protein bands of the same MW, indicating that the differential migration patterns between the WT and the *alg3Δ* mutant were caused by differential *N*-glycosylation (Fig. 3A, left panel). Lectin blotting using Galanthus nivalis agglutinin, which detects terminal α1,2-, α1,3-, and α1,6-linked mannose residues, showed the decrease in the overall size of the secretory proteins from the *alg3Δ* mutant compared to the WT even more clearly (Fig. 3A, right panel). After PNGase F treatment, the WT and *alg3Δ* strains produced protein bands with the same MW in a lectin blot, indicating that most of the secretory proteins were *N*-glycosylated proteins.

We investigated the effect of *ALG3* deletion on the glycoproteins phospholipase Plb1 and T-cell antigen MP98, which were predicted to contain 15 and 10 *N*-glycosylation sites, respectively. To analyze the glycosylation patterns of Plb1 and MP98, His-tagged and glycosylphosphatidylinositol (GPI)-anchorless Plb1 and MP98 were expressed in various glycosylation mutant strains (Fig. 3B and C). Plb1 proteins secreted from the *alg3Δ* mutant and from the *och1Δ* and *mnn2Δ N*-glycosylation mutants, which are defective in elongation of the mannose outer chains of *N-*glycans (30), had lower MWs than those from the WT strain (Fig. 3B). Because MP98 was predicted to have *N*- and *O*-glycosylation sites in its C-terminal Ser/Thr-rich region, the migration pattern of MP98 was also investigated in *ktr3Δ* and *hoc3Δ* mutants, which are defective in the biosynthesis of major *O*-linked glycans (32). The MP98 protein bands of the *alg3Δ* mutant migrated substantially faster than those of the WT strain, reflecting a size decrease due to the attachment of truncated *N*-glycans, whereas no apparent change in migration was detected in the *O*-glycosylation mutants (Fig. 3C). These results strongly indicated that both Plb1 and MP98 are highly modified by *N*-glycosylation, which is clearly defective in the *alg3Δ* strain.

### The *alg3Δ* mutant displays moderately defective virulence-associated phenotypes *in vitro*.

**FIG 4:**
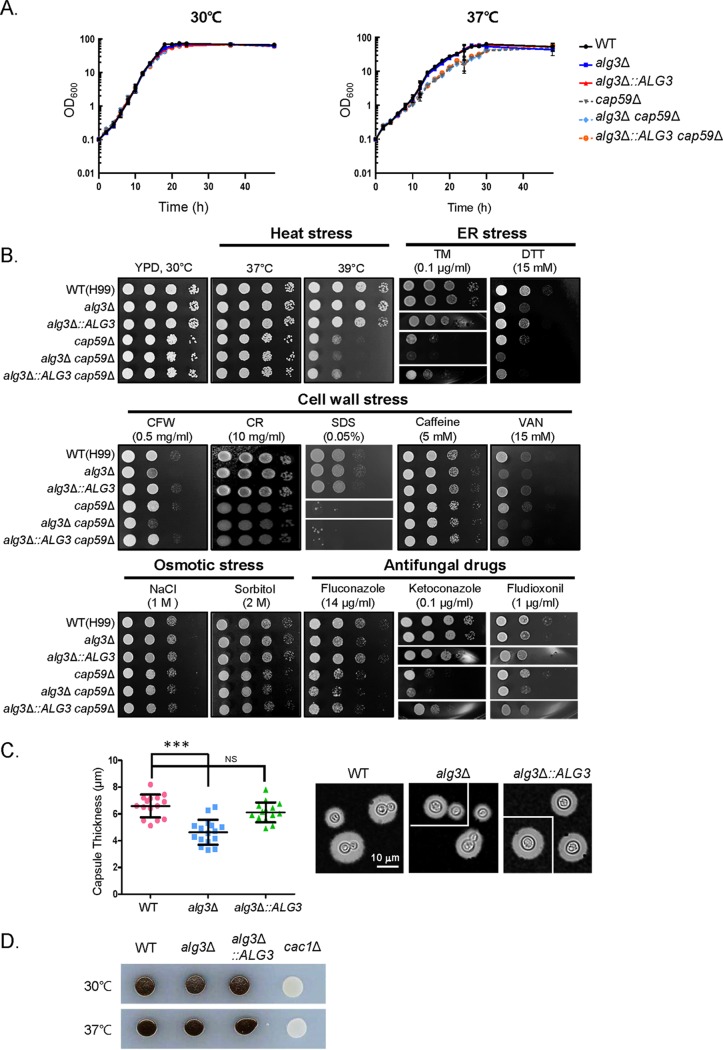
*In vitro* analysis of virulence-associated phenotypes of C. neoformans *alg3*Δ. (A) Growth analysis of C. neoformans strains cultivated in liquid YPD medium. C. neoformans cells of capsule background strains (WT, *alg3*Δ, and *alg3*Δ::*ALG3*) and acapsular background strains (*cap59*Δ, *alg3*Δ *cap59*Δ, and *alg3*Δ::*ALG3 cap59*Δ) were incubated in shaker flasks containing YPD media at 30°C and 37°C. Data represent means of results from two independent C. neoformans cultures. (B) Spotting analysis of growth of C. neoformans WT, *alg3*Δ, *alg3*Δ::*ALG3*, *cap59*Δ, *alg3*Δ *cap59*Δ, and *alg3*Δ::*ALG3 cap59*Δ strains under conditions of heat stress (37°C or 39°C), ER stress (TM, tunicamycin; DTT, dithiothreitol), cell wall stress (CFW, calcofluor white; CR, Congo red; VAN, vanadate), osmotic stress (NaCl or sorbitol) and antifungal drug treatments (fluconazole, ketoconazole, or fludioxonil). The spotting data from non-adjacent lines on the same plate are indicated by the white spaces between the data lanes. (C) Analysis of capsule formation. ***, *P < *0.0001 (WT versus *alg3*Δ mutant); NS, not significant (WT versus *alg3*Δ::*ALG3* mutant). The images from representative C. neoformans cells located in non-contiguous portions of the same photo are indicated by the white lines between images. (D) Analysis of melanin synthesis in the indicated strains. The *cac1*Δ strain was included as a control strain showing defective melanization on l-DOPA medium.

Growth of the *alg3Δ* mutant was nearly equivalent to that of the WT under normal growth conditions (YPD [1% {wt/vol} yeast extract, 2% {wt/vol} peptone, 2% {wt/vol} dextrose], 30°C) and even under heat stress conditions (YPD, 37°C) in liquid cultivation (Fig. 4A), indicating that the defective lipid-linked *N*-glycan assembly caused by *ALG3* deletion does not affect normal growth of C. neoformans. Considering that cell wall defects caused by *ALG3* deletion might be masked by the polysaccharide capsule that covers the cell walls in C. neoformans, we constructed capsule-deficient strains by deleting *CAP59*, which reportedly is involved in capsule biosynthesis (33). Absence of capsule did not affect the growth of the *alg3Δ* mutant at either 30°C or 37°C (Fig. 4A), although the growth of the C. neoformans cells in the acapsular background was slightly inhibited at 37°C (Fig. 4A, right panel). We compared the growth phenotypes of the WT and *alg3Δ* mutant strains under various stress conditions, including ER, cell wall, and osmotic stresses, which are closely associated with the virulence of C. neoformans. The *alg3Δ* mutant showed moderately decreased growth in the presence of an ER stress-inducing reagent, dithiothreitol (DTT), and in the presence of the cell wall stressors calcofluor white (CFW) and vanadate (VAN) (Fig. 4B). Growth of the *alg3Δ* mutant was slightly decreased upon fluconazole treatment. However, the *alg3Δ* mutant did not show increased sensitivity to the other ER stress reagent, tunicamycin (TM), to other cell wall stressors, including Congo red (CR), sodium dodecyl sulfate (SDS), and caffeine; to osmotic stressors, including NaCl and sorbitol; or to antifungal reagents such as ketoconazole and fludioxonil (Fig. 4B). This indicated that loss of Alg3 function does not markedly affect the ability of C. neoformans to cope with diverse stresses. Growth of the acapsular *cap59Δ* strain was inhibited at 39°C and in the presence of SDS. *ALG3* deletion moderately increased the sensitivity of the *cap59Δ* strain to TM, DTT, CFW, and antifungal drugs but did not affect the sensitivity to high temperature, CR, caffeine, and osmotic stressors such as NaCl and sorbitol. These observations suggested that even in the absence of a capsule, the defective core *N*-glycan structure did not markedly affect C. neoformans growth under most culture conditions (Fig. 4B).

Next, we investigated the effect of defective core *N*-glycan assembly on the production of two major virulence factors, antioxidant melanin and antiphagocytic capsule. The capsule thickness of the *alg3Δ* mutant was decreased by only ~15% compared to the WT and the *alg3Δ*::*ALG3* complemented strains, indicating a minor role of Alg3 in capsule formation (Fig. 4C). Furthermore, the *alg3Δ* mutant produced WT levels of melanin on l-3,4-dihydroxyphenylalanine (l-DOPA) medium, indicating that Alg3 is dispensable for melanin synthesis (Fig. 4D). This is consistent with previously reported characteristics of a C. neoformans *alg3* mutant strain, generated by inactivation of a CNAG_05142 allele through random insertional mutagenesis, which also displayed normal melanin production without detectable growth defects on cell wall stress media (34). Collectively, the results strongly indicated that defective *N*-glycosylation caused by *ALG3* deletion has minimal effects on the biosynthesis of certain key virulence factors.

### *ALG3* deletion abolishes virulence in C. neoformans.

**FIG 5:**
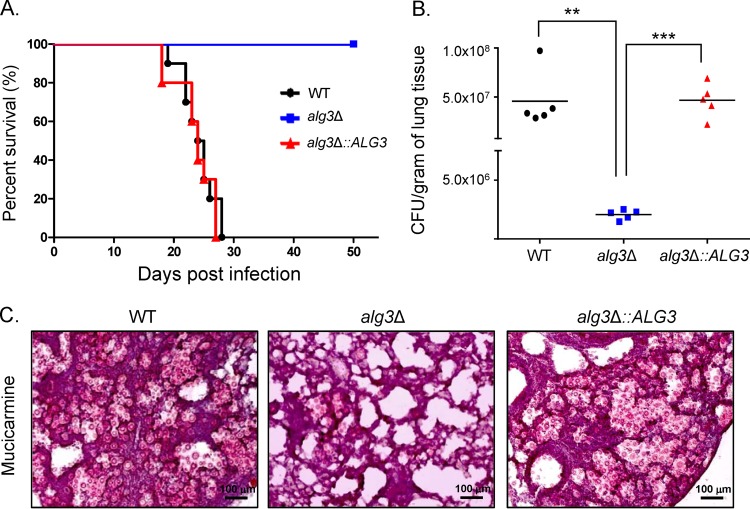
*In vivo* analysis of virulence of C. neoformans *alg3*Δ. (A) A/Jcr mice were infected with 10^5^ cells of WT, *alg3*Δ, or *alg3*Δ::*ALG3* cells by intranasal instillation. Percent survival was monitored for 7 weeks postinfection. (B) CFU counts per gram of lung tissue were determined upon sacrifice of the mice infected with WT, *alg3*Δ, or *alg3*Δ::*ALG3* cells. **, *P < *0.05 (WT versus *alg3*Δ mutant); ***, *P < *0.0005 (*alg3*Δ mutant versus *alg3*Δ::*ALG3* mutant) (one-way analysis of variance and Bonferroni *post hoc* tests). (C) Histopathological staining of infected lung tissues with mucicarmine. Lung tissues were obtained from infected mice at day 7.

To assess the impact of truncated core *N*-glycans on pathogenicity, we analyzed the virulence of the *alg3Δ* strain in a murine model of systemic cryptococcosis established by intranasal infection. In contrast to the moderate defects in virulence-associated phenotypes *in vitro*, the *alg3Δ* strain was avirulent, whereas the *alg3Δ*::*ALG3* complemented strain was as virulent as the WT strain (Fig. 5A). Even at 50 days postinfection (dpi), none of the mice infected with the *alg3Δ* strain showed signs of pain or illness, indicating that the intact core *N*-glycan structure is critical for virulence in C. neoformans. Examining fungal burden in the lungs at 7 dpi, we found that the *alg3Δ* mutant showed a marked decrease in lung colonization compared to the WT and *alg3*Δ::*ALG3* strains (Fig. 5B). Histopathological analysis of mucicarmine-stained polysaccharide capsule of C. neoformans revealed extensive proliferation of the WT and *alg3*Δ::*ALG3* cells, with huge capsules in the lung tissues, and minimal presence of *alg3*Δ cells, which was consistent with the fungal burden data (Fig. 5C), strongly suggesting that the *alg3Δ* mutant did not proliferate in the lungs following pulmonary infection. These results implied that *ALG3* deletion has a major impact on the growth of C. neoformans *in vivo* or on its interaction with the host immune system.

### *ALG3* deletion does not impair the early stages of host interaction in pulmonary infection.

**FIG 6:**
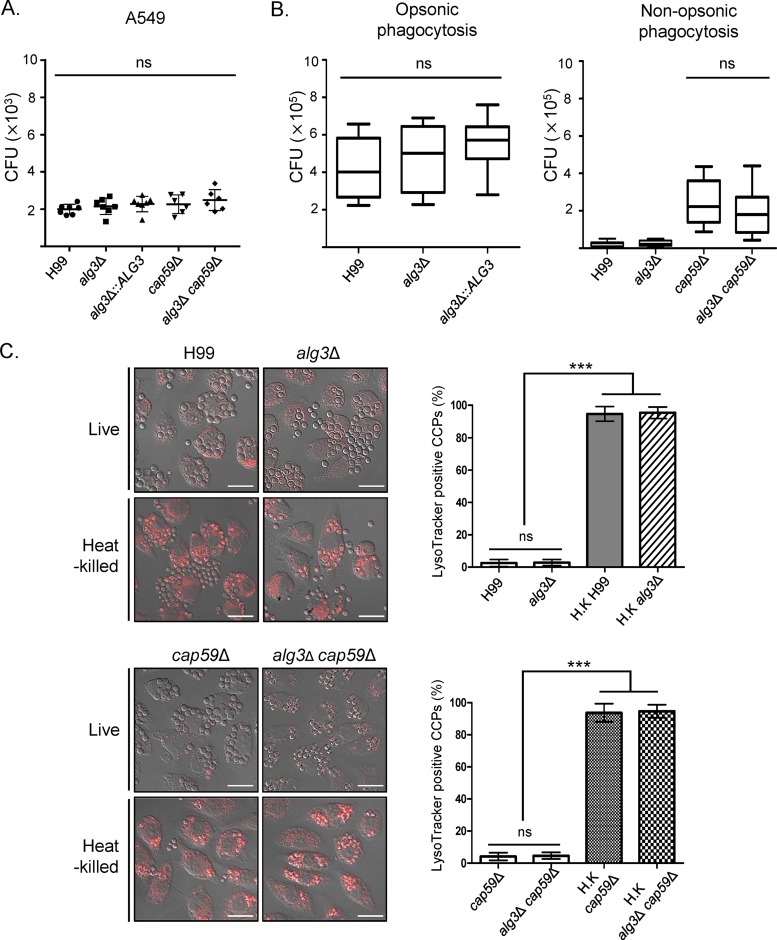
*In vitro* analysis of the interaction of C. neoformans *alg3*Δ with host cells. (A) Analysis of adherence to lung epithelial cells. CFU of C. neoformans cells recovered from host cell lysates were counted after coincubation of C. neoformans cells with A549 lung epithelial cells at 37°C with 5% CO_2_ for 1 h. (B) Analysis of phagocytosis with or without opsonization. C. neoformans cells were incubated with activated J774A.1 macrophages for 2 h. CFU were counted to determine opsonic and nonopsonic phagocytosis. NS, not significant for all cells (determined by one-way analysis of variance and Bonferroni *post hoc* tests). (C) Analysis of phagosome acidification. Live and heat-killed C. neoformans cells were incubated with activated macrophage (J774A.1) for 2 h. Staining with LysoTracker Red was used to detect acidic phagosomes, and heat-killed C. neoformans cells served as a positive control for phagosomal acidification. The microscopy images, which overlap the confocal images, were obtained by differential interference contrast (DIC). Representative images are presented with scale bars (20 μm). The percentage of acidified macrophages among *Cryptococcus*-containing phagolysosomes (CCPs) was calculated by counting the number of LysoTracker Red-stained phagolysosomes and dividing that number by the number of CCPs. Data representing means ± standard deviations were generated from three biologically independent experiments with at least 100 CCPs per infection. Statistical analysis was performed using an unpaired two-tailed Student's *t* test (ns, not significant; ***, *P < *0.0001).

Glycans assembled on cell surface glycoproteins of fungal pathogens affect the efficiency of host cell interaction during infection. As *Cryptococcus* cells are exposed first to host lung cells after respiratory infection, we compared the efficiencies of adhesion to A549 lung epithelial cells between WT cells and *alg3Δ* mutant cells. No significant difference was observed in adhesion efficiency between the WT cells and the *alg3Δ* mutant cells, indicating that the altered core *N*-glycan structure did not affect the interaction of fungal cells with lung epithelial cells (Fig. 6A). To evaluate potential differences between the WT and *alg3Δ* strains in subsequent infection steps, we conducted opsonic and nonopsonic phagocytosis assays by incubating cryptococcal cells with macrophage-like cells (J774A.1) for 2 h. The numbers of CFU of *alg3Δ* cells recovered upon opsonic and nonopsonic phagocytosis were comparable to those seen with the WT, indicating that phagocytosis was not compromised by truncated core *N*-glycans (Fig. 6B). Accordingly, the numbers of C. neoformans cells engulfed inside macrophage cells were very similar among the tested strains (Fig. S3A and B).

10.1128/mBio.00711-20.4 FIG S3 Opsonic and nonopsonic phagocytosis. (A) Encapsular C. neoformans cells of the WT, *alg3*Δ, and *alg3*Δ::*ALG3* strains were opsonized with 18B7 antibody and incubated with J774A.1 cells (C. neoformans/J774A.1 ratio, 10:1) for 2 h. Then, nonphagocytized yeast cells were removed by washing, and phagocytized yeast cells were evaluated by microscopy. (B) Acapsular WT, *alg3*Δ, *cap59*Δ, and *alg3*Δ *cap59*Δ yeast cells were incubated with macrophages (C. neoformans/J774A.1 ratio, 10:1) for 2 h. Nonphagocytized yeast cells were removed, and phagocytized yeast cells were evaluated under a Zeiss confocal microscope. Scale bars, 20 μm. Download FIG S3, PDF file, 0.4 MB. Copyright © 2020 Thak et al. 2020 Thak et al. This content is distributed under the terms of the Creative Commons Attribution 4.0 International license.

Following phagocytosis, phagosome maturation is critical for the clearance of pathogen cells. However, C. neoformans manipulates phagosome maturation by blocking phagosome acidification to avoid intracellular killing by host phagocytes (35, 36). Thus, we compared the abilities of the WT and *alg3Δ* strains to block phagosome acidification by staining macrophages incubated with live and heat-killed C. neoformans cells for 2 h with the acidotropic dye LysoTracker Red. The ability of the *alg3Δ* strain to block phagosomal acidification was comparable to that of the WT (Fig. 6C). Whereas the phagosomes containing heat-killed WT cells were mostly acidified regardless of the presence or absence of capsule (H99 and *cap59Δ* strains), most phagosomes containing live C. neoformans cells did not acidify (Fig. 6C, left panels). Notably, both the *alg3Δ* and *alg3Δ cap59Δ* strains exhibited an ability to inhibit phagosomal acidification comparable to that exhibited by the WT strain (Fig. 6C, right panels). These results indicated that, despite its compromised virulence, the *alg3Δ* mutant retains the ability to modify host phagosome maturation.

### Truncated core *N*-glycans are defective in inducing macrophage death after phagocytosis.

**FIG 7:**
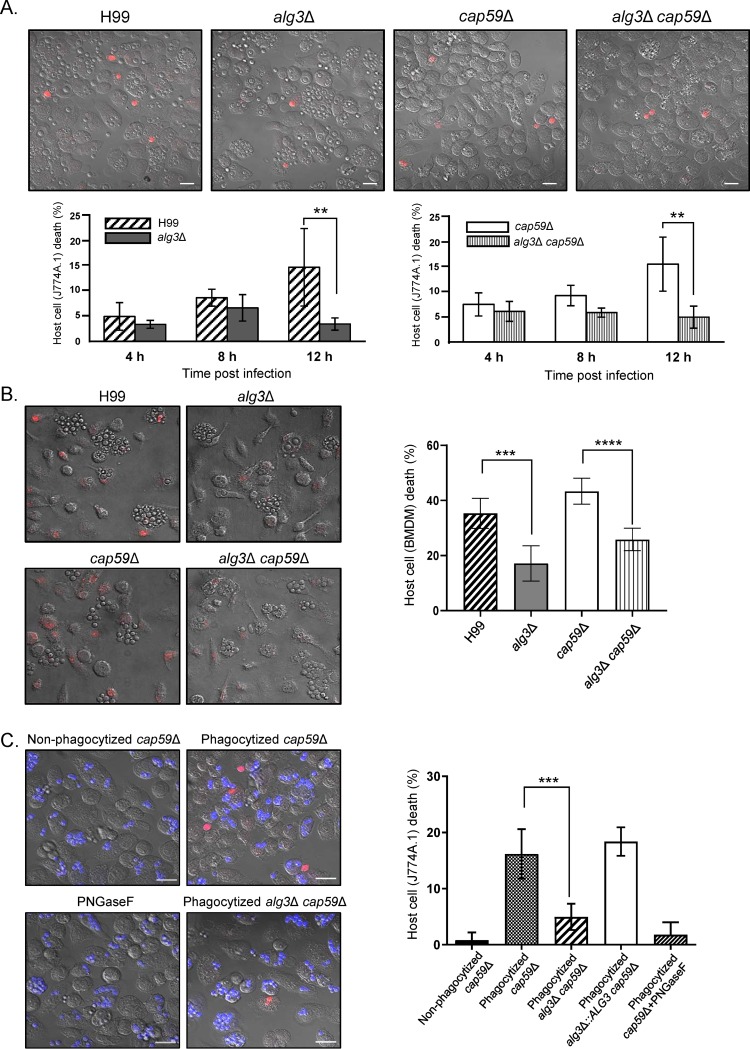
C. neoformans *alg3*Δ cells are defective in inducing macrophage cell death after phagocytosis. (A) Macrophage cell death induced by C. neoformans infection with or without opsonization. Activated J774A.1 macrophage cells were coincubated with C. neoformans and stained with propidium iodide (PI; red) at 4 h, 8 h, and 12 h postinfection. Representative images taken 12 h postinfection are presented. The statistical analysis was performed using an unpaired two-tailed Student’s *t* test (**, *P* < 0.01). (B) BMDM cell death induced by C. neoformans infection with or without opsonization. Before coincubation with C. neoformans was performed, BMDM cells were primed with lipopolysaccharide (LPS) (100 ng/ml) for 3 h. Dead macrophages were stained with PI at 12 h postinfection. Representative images taken 12 h postinfection are presented. Data represent means ± standard deviations of results determined for 100 to 200 phagosomes from three biologically independent experiments. The statistical analysis was performed using an unpaired two-tailed Student’s *t* test (***, *P* < 0.001; ****, *P* < 0.0001). (C) Macrophage cell death induced by heat-killed C. neoformans cells after preexposure to macrophages (J774A.1). The C. neoformans cells were stained with calcofluor white (blue) for identification of fungal cells in macrophages, and lysed macrophages were stained with PI to identify dead host cells. Macrophage cell death events were imaged at 4 h postinfection. The microscopy images, which overlap the confocal images, were obtained by DIC. Scale bars, 20 μm. Data represent means ± standard deviations of results from three biologically independent experiments, *n* =100 infection events. Statistical analysis was performed using an unpaired two-tailed Student's *t* test (ns, not significant; ***, *P < *0.0001).

It was previously proposed that intracellular fungal pathogens, such as C. albicans and C. neoformans, exploit macrophage pyroptosis, a host cell death program induced via fungal cell wall remodeling in response to the macrophage phagosome, as a mechanism of host escape (21, 37, 38). In particular, exposure of glycosylated proteins by cell wall remodeling was shown to be sufficient to activate macrophage lysis without requiring fungal cell viability (38). Therefore, we examined whether the C. neoformans *alg3Δ* mutant was defective in triggering *in vitro* macrophage cell death (Fig. 7A). We incubated J774A.1 macrophages with C. neoformans WT, *alg3Δ*, *cap59Δ*, and *alg3Δ cap59Δ* cells for 4 h, 8 h, and 12 h and quantified macrophage cell death by staining them with propidium iodide (PI). Whereas no significant difference was observed between the WT and *alg3Δ* strains in both the encapsular and acapsular backgrounds at 4 h and 8 h postincubation, the *alg3Δ* mutant induced clearly reduced macrophage lysis at 12 h (Fig. 7A). To validate the C. neoformans-induced host cell death, observed with the J774A.1 macrophage-like cell lines, in the primary host immune cell lines, we cocultured bone marrow-derived macrophages (BMDMs) with the WT, *alg3*Δ, *cap59*Δ, and *alg3*Δ *cap59*Δ strains. A reduction of about 50% in PI staining was observed in BMDMs infected by the *alg3*Δ mutant compared to BMDMs infected by the WT strain (Fig. 7B). The results strongly support the idea that the macrophage cell death induced by C. neoformans infection was defective in the *alg3*Δ mutant carrying the truncated core *N*-glycans (Fig. 7B).

To assess whether the truncated core *N*-glycans assembled on the cell wall mannoproteins (cwMPs) of the *alg3Δ* strain contribute to the defect in triggering macrophage cell death after phagocytosis, as previously proposed (38), prephagocytized and nonphagocytized *cap59Δ* and *alg3Δ cap59Δ* cells were heat-killed and inoculated with J774A.1 macrophages (Fig. 7C). The prephagocytized *cap59Δ* cells clearly primarily generated dead cells that were stained in red by PI. In contrast, we observed a very low frequency of macrophage lysis upon infection with the nonphagocytized C. neoformans cells, which might have mostly contained extracellular C. neoformans that did not go through phagocytosis and a small portion of C. neoformans cells that were released via vomocytosis. This is consistent with a previous report that the capacity of killed fungal cells to drive macrophage lysis requires cell wall remodeling in the macrophage phagosome environment (38). Notably, the *alg3Δ cap59Δ* strain showed a markedly reduced macrophage lysis rate, indicating that truncated core *N*-glycans are defective in inducing macrophage cell death. The *alg3Δ*::*ALG3* strain constructed in *cap59Δ* background, which showed recovered growth under stress conditions compared to the *alg3Δ cap59Δ* strain (Fig. 4B), induced macrophage cell death at a rate comparable to that seen with the WT strain (Fig. 7C), indicating that the reduction in macrophage lysis was caused by the loss of Alg3 function. The phagocytized *cap59*Δ cells lacking *N*-glycans, generated by PNGase F treatment, showed a lower lysis rate than the nontreated phagocytized *cap59*Δ cells (Fig. 7C), supporting the idea of the critical role of *N*-glycans as a cell surface moiety in inducing macrophage cell death.

### Cell wall remodeling in the phagosome environment is not defective in the *alg3Δ* strain.

**FIG 8:**
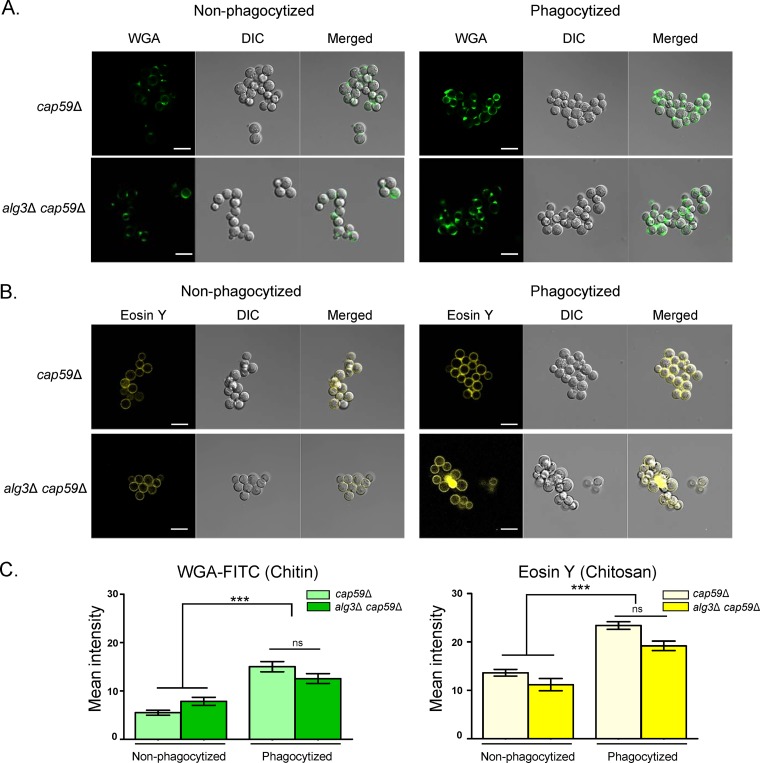
Truncated core forms of *N*-glycans do not affect cell wall remodeling after phagocytosis. (A and B) C. neoformans cells were incubated with macrophages for 4 h. Nonphagocytized and phagocytized cells were stained with fluorescein isothiocyanate-conjugated wheat germ agglutinin (FITC-WGA) to visualize exposed chitin (A) and with eosin Y to visualize chitosan (B). Morphologies of yeast cells were visualized using DIC microscopy. Scale bars, 10 μm. (C) Quantification of FITC-WGA and eosin Y staining intensities. Average fluorescence intensity of at least 50 individual cryptococcal cells was analyzed using NIS-Elements (Nikon). Statistical analysis was performed using an unpaired two-tailed Student's *t* test (***, *P < *0.0001).

Fungal pathogens modify their cell wall composition or structure upon environmental changes (39, 40). Exposure of fungal cell surface glycoproteins via cell wall remodeling in the macrophage phagosome environment is required to induce macrophage lysis (38). To examine whether the defect in triggering macrophage cell death in the *alg3Δ* mutant results from an inability to undergo cell wall remodeling after phagocytosis, we stained chitin and chitosan, which are the innermost components of C. neoformans cell walls. In agreement with a previous report that *ALG3* mutation does not affect cell wall content (34), we observed that the *alg3Δ* and WT strains displayed similar staining intensities for exposed chitin (Fig. 8A) and chitosan (Fig. 8B) under normal culture conditions. Moreover, the WT and *alg3*Δ strains exhibited similar increases in levels of chitin and chitosan after phagocytosis, indicating that loss of Alg3 function does not affect the capacity for cell wall remodeling after phagocytosis (Fig. 8C). These results strongly indicated that the defect of truncated core *N*-glycans in inducing macrophage cell death was not caused by defective cell wall remodeling but might have been related to the generation of a signal to host cells for induced cell death.

### Intact core *N*-glycans are required for macrophage cell death and full virulence of C. neoformans.

**FIG 9:**
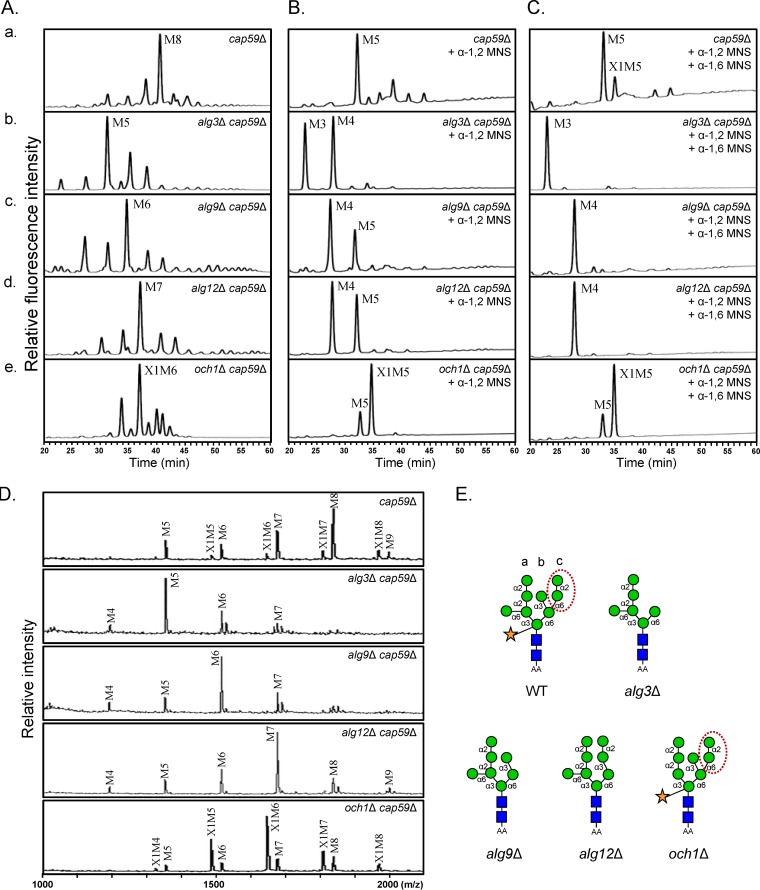
Structural analysis of truncated core *N*-glycans generated by C. neoformans *alg9*Δ and *alg12*Δ mutants. (A) HPLC profiles of neutral *N*-glycans of cwMPs from the *cap59*Δ, *alg3*Δ *cap59*Δ, *alg9*Δ *cap59*Δ, *alg12*Δ *cap59*Δ, and *och1*Δ *cap59*Δ mutant strains. (B) HPLC profiles of neutral *N*-glycans after α-1,2 mannosidase treatment. (C) HPLC profiles after serial treatment of neutral *N*-glycans with α-1,2 mannosidase and α-1,6 mannosidase. (D) MALDI-TOF profiles of neutral *N*-glycans. AA-labeled neutral *N*-glycans of the *cap59*Δ, *alg3*Δ *cap59*Δ, *alg9*Δ *cap59*Δ, *alg12*Δ *cap59*Δ, and *och1*Δ *cap59*Δ mutant strains were selectively collected and analyzed by MALDI-TOF. (E) The representative structure of the major *N*-glycan species generated by each strain is presented in red-dotted ovals with an indication of the upper-arm α-1,2-Man-α-1,6-Man-residues of branch c, which are missing in *alg* mutant-generated *N*-glycans.

To define the functional structure of core *N*-glycans required for the full induction of host cell death and virulence, we further deleted the *ALG9* and *ALG12* genes, which are involved in lipid-linked *N*-glycan biosynthesis downstream of Alg3 (Fig. 1A). Following the addition of α-1,3 mannose to Dol-PP-GlcNAc_2_Man_5_ by Alg3, an α-1,2-linked mannose was added by the α-1,2-mannosyltransferase Alg9, followed by the addition of an α-1,6-mannose by the α-1,6-mannosyltransferase Alg12. Then, the α-1,6-mannose branch was capped with an α-1,2-linked mannose residue added by Alg9 (Fig. 1A). Thus, Alg9 has a dual function in lipid-linked *N*-glycan biosynthesis (31). S. cerevisiae *alg9*Δ and *alg12*Δ mutants accumulate GlcNAc_2_Man_6_ and GlcNAc_2_Man_7_, respectively, as major *N*-glycan species attached to glycoproteins (41, 42). Bioinformatics analysis suggested that CNAG_05881 encodes C. neoformans Alg9, which is predicted to have 7 transmembrane domains and a glycosyltransferase 22 family domain (Fig. S4A). Intriguingly, two putative genes, CNAG_02715 and CNAG_07527, were annotated to encode Alg12. Both the CNAG_02715 and CNAG_07527 proteins also had multiple transmembrane domains and a glycosyltransferase 22 family domain (Fig. S4B). We constructed mutant strains with deletion of CNAG_05881, CNAG_02715, and CNAG_07527, respectively (Fig. S5A), and analyzed their *N*-glycan profiles. HPLC analysis of neutral *N*-glycans revealed that deletion of CNAG_05881 and CNAG_02715 resulted in the accumulation of truncated core *N*-glycans with six (M6) and seven (M7) mannose residues as major *N*-glycan species, respectively (Fig. 9A), supporting the idea that CNAG_05881 and CNAG_02715 encode functional Alg9 and Alg12, respectively, and are involved in core *N*-glycan assembly in the ER of C. neoformans. However, deletion of CNAG_07527 did not affect the *N*-glycan profile (Fig. S5B), indicating that CNAG_07527 protein might have other functions beside *N*-glycan biosynthesis. Treatment with α-1,2 and α-1,2/α-1,6 mannosidases further indicated that the *alg9*Δ *cap59*Δ and *alg12*Δ *cap59*Δ strains produced truncated core *N*-glycans with or without α-1,6 outer mannose chains, in contrast to the *och1*Δ *cap59*Δ strain, which produced intact core *N*-glycans without outer mannose chains (Fig. 9B and C). In addition, as observed for the *alg3*Δ *cap59*Δ mutant, no xylose-containing M5 (X1M5) peak was detected in the *N-*glycan profiles of the *alg9*Δ *cap59*Δ and *alg12*Δ *cap59*Δ strains after sequential exomannosidase treatment, in contrast to those of the WT and *och1*Δ *cap59*Δ strains (Fig. 9C). The lack of xylose residues in the *N-*glycans of the *alg9*Δ *cap59*Δ and *alg12*Δ *cap59*Δ strains was further confirmed by matrix-assisted laser desorption ionization–time of flight (MALDI-TOF) analysis (Fig. 9D). These data indicated that truncated core *N*-glycans generated by *alg9*Δ and *alg12*Δ cells were extended through addition of α-1,2 mannose residues and a single α-1,6 outer chain mannose, whereas xylose residues were nearly completely lacking (Fig. 9E).

10.1128/mBio.00711-20.5 FIG S4 Bioinformatics analysis of Alg9 and Alg12. (A and B) Predicted transmembrane and Glyco_transf_22 (glycosyltransferase 22 family) domains of Alg proteins are indicated by slashes and dotted boxes, respectively. Data represent sequence identities and similarities between Alg9 and Alg12 homologs of S. cerevisiae, H. polymorpha, C. albicans, A. niger, and S. pombe. Multiple-sequence alignments of Alg9 and Alg12 homologs were generated using ClustalW 1.8. Identical residues and conservative amino acid substitutions are indicated by black and gray shading, respectively. Download FIG S4, PDF file, 0.2 MB. Copyright © 2020 Thak et al. 2020 Thak et al. This content is distributed under the terms of the Creative Commons Attribution 4.0 International license.

10.1128/mBio.00711-20.6 FIG S5 Disruption of *ALG9* and *ALG12*. (A) Strategy for CNAG_05881 and CNAG_07527 disruption using the NAT split marker and CNAG_02715 disruption using the neomycin (*NEO*) split marker. Stable transformants were selected on YPD medium containing nourseothricin (100 μg/ml) and neomycin (200 μg/ml), respectively, and were screened by PCR. (B) HPLC-based neutral glycan profiles of *cap59*Δ, *alg9*Δ *cap59*Δ, and *alg12*Δ *cap59*Δ strains. Download FIG S5, PDF file, 0.2 MB. Copyright © 2020 Thak et al. 2020 Thak et al. This content is distributed under the terms of the Creative Commons Attribution 4.0 International license.

**FIG 10:**
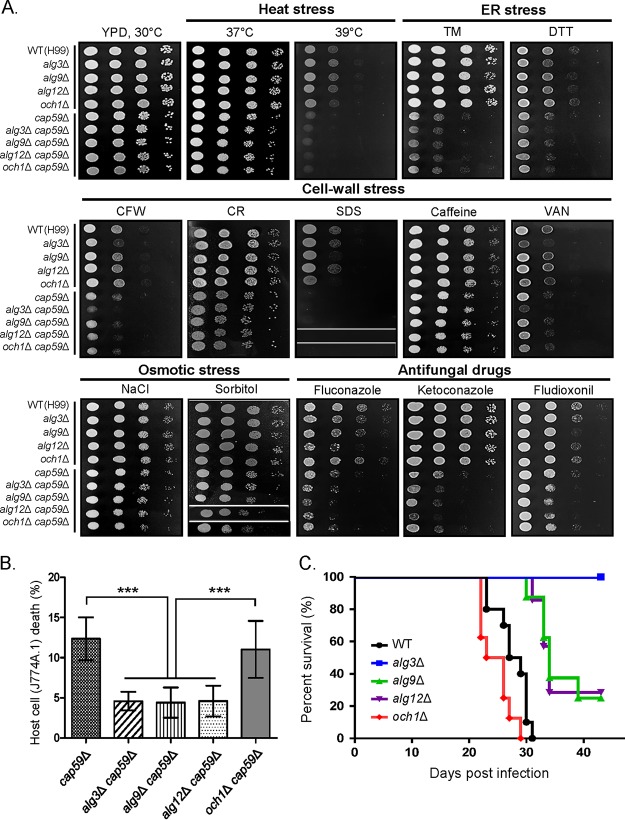
Analysis of growth and virulence-associated phenotypes of C. neoformans *alg9*Δ and *alg12*Δ. (A) Spotting analysis of growth of the C. neoformans WT, *alg3*Δ, *alg9*Δ, *alg12*Δ, *och1*Δ strains under conditions of heat stress (37 or 39°C), ER stress (TM or DTT), cell wall stress (CFW, CR, SDS, caffeine, or VAN), osmotic stress (NaCl or sorbitol), and treatment with antifungal drugs (fluconazole, ketoconazole, or fludioxonil). The spotting data from non-adjacent lines on the same plate are indicated by the white spaces between the data lanes. (B) Macrophage cell death induced by heat-killed C. neoformans *cap59*Δ, *alg3*Δ *cap59*Δ, *alg9*Δ *cap59*Δ, *alg12*Δ *cap59*Δ, or *och1*Δ *cap59*Δ cells after preexposure to macrophages. Statistical analysis was performed using an unpaired two-tailed Student’s *t* test (***, *P* < 0.0001). (C) *In vivo* analysis of virulence of C. neoformans *N*-glycan mutant strains. A/Jcr mice were infected with 10^5^ cells of the WT, *alg3*Δ, *alg9*Δ, *alg12*Δ, or *och1*Δ strain by intranasal instillation. Percent survival was monitored for 7 weeks postinfection.

We compared the levels of growth of *alg* null mutant strains under various stress conditions and in the presence of antifungal reagents (Fig. 10A). Overall, *alg9*Δ and *alg12*Δ cells did not show retarded growth under the tested stress conditions, with the exception that the *alg9*Δ and *alg12*Δ cells showed retarded growth in the presence of SDS and fluconazole, respectively. Notably, all *alg* mutants in the acapsular background showed decreased growth in the presence of fluconazole, ketoconazole, or fludioxonil compared to the *cap59*Δ strain. In contrast, sensitivity to various stresses was not increased in the *och1*Δ *cap59*Δ strain compared to that in the *cap59*Δ strain (Fig. 10A). Notably, the *alg9*Δ *cap59*Δ and *alg12*Δ *cap59*Δ strains also showed remarkably reduced macrophage cell death activity compared to the *alg3*Δ *cap59*Δ strain, whereas the *och1*Δ *cap59*Δ strain retained the capacity to induce macrophage cell death, with an efficiency comparable to that of the WT strain (Fig. 10B). The complemented *alg9*Δ::*ALG9* strain (Fig. S6A) and *alg12*Δ::*ALG12* strain (Fig. S6B) were constructed and tested for growth recovery under various stress conditions (Fig. S6C). The complemented strains completely recovered the growth inhibition by antifungal reagents and *N*-glycan profiles as analyzed by HPLC (Fig. S6D).

10.1128/mBio.00711-20.7 FIG S6 Construction of *ALG9* and *ALG12* complementation strain. (A) Strategy for complementary CNAG_05881 using the *NEO* split marker. Stable transformants were selected on YPD medium containing *NEO*. (B) Strategy for complementary CNAG_02715 using the NAT split marker. Stable transformants were selected on YPD medium containing NAT. (C) Spotting analysis of growth recovery of C. neoformans *alg9*Δ::*ALG9* and *alg12*Δ::*ALG12* strains under conditions of heat stress, ER stress, cell wall stress, osmotic stress, and antifungal drug treatments. (D) HPLC-based neutral glycan profiles of H99, *alg9*Δ, *alg9*Δ::*ALG9* 1, *alg12*Δ, and *alg12*Δ::*ALG12* 2 strains. Download FIG S6, PDF file, 0.3 MB. Copyright © 2020 Thak et al. 2020 Thak et al. This content is distributed under the terms of the Creative Commons Attribution 4.0 International license.

To investigate the effect of truncated core *N*-glycans on *in vivo* pathogenicity, we analyzed the virulence of the *alg9Δ* and *alg12Δ* mutants, along with the WT, *alg3Δ*, and *och1Δ* strains, in a murine model of systemic cryptococcosis. The *alg9Δ* and *alg12Δ* strains were apparently less virulent than the WT and *och1Δ* strains, but they partly retained the pathogenic activity, in contrast to the *alg3Δ* mutant, which showed almost completely abolished virulence (Fig. 10C), which might reflect the relatively higher level of growth of the *alg9Δ* and *alg12Δ* strains than of the *alg3Δ* strain under stress conditions. These results strongly indicate that the intact core *N*-glycan structure is a crucial factor in modulating host cell death and thus determining pathogenicity of C. neoformans.

### Survival within macrophage and immune response activation were decreased in the *alg3Δ* mutant strain.

**FIG 11:**
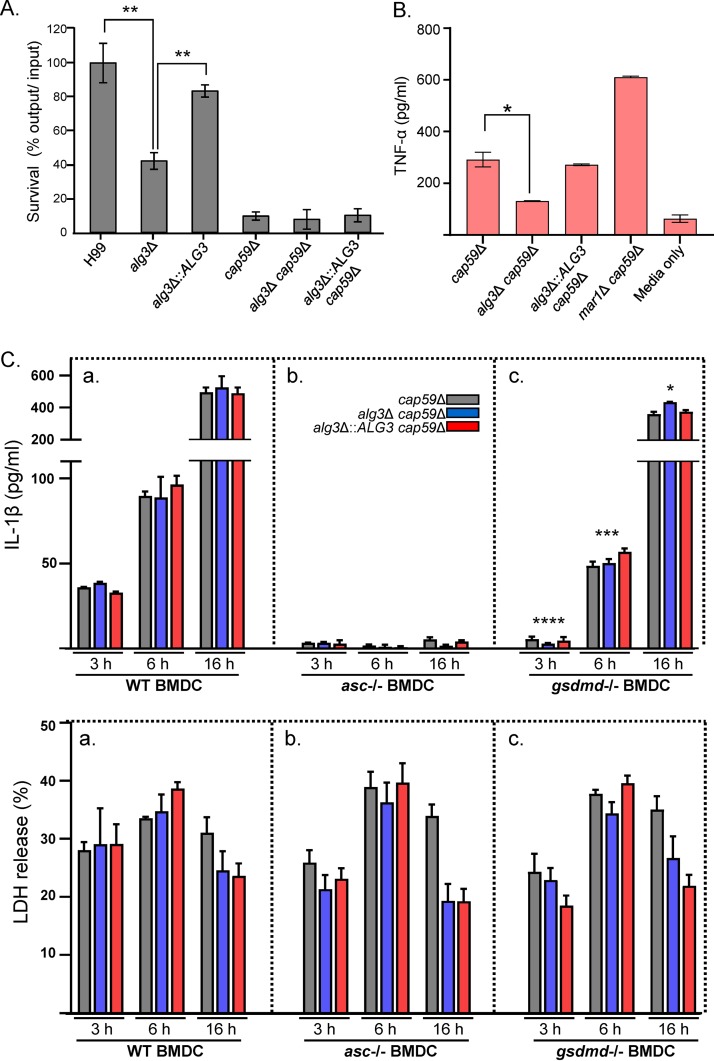
Analysis of interaction of *alg3Δ* cells with the primary host immune cells. (A) Survival rate in macrophages. Killing of C. neoformans by BMDMs was determined through colony formation derived from lysed macrophages. The average percentages representing the input/output ratios of the cells were determined by counting CFU with three biologically independent experiments. (B) Analysis of host immune response activation. TC-cultured C. neoformans cells were cocultivated with BMDCs, and the levels of TNF-α secreted from dendritic cells were measured. All the statistical analyses were performed using an unpaired two-tailed Student's *t* test (*, *P* < 0.05; **, *P* < 0.001). (C) Pyroptosis analysis. BMDCs from WT, *Asc*^−/−^, and *Gsdmd*^−/−^ mice were infected with cells of the *cap59*Δ, *alg3*Δ *cap59*Δ, and *alg3*Δ::*ALG3 cap59*Δ acapsular background strains for the indicated times (3 h, 6 h, and 16 h). The levels of IL-1β secreted from the cell culture supernatant were assayed via ELISA (*, *P < *0.05; ***, *P < *0.0005; ****, *P < *0.0001 [WT versus *Gsdmd*^−/−^ BMDCs]).

Decreased host cell death, which is exploited by C. neoformans to escape host cells, would generate prolonged retention of pathogens in host immune cells, resulting in a reduced ability of the *alg3*Δ mutant to survive and proliferate within macrophages. We compared the survival rates of C. neoformans WT and *alg3*Δ mutant strains in BMDM after for 24 h of infection. It was observed that the *alg3*Δ mutant displayed an apparent (∼40%) reduction of its ability to survive in macrophages compared to the WT strain (Fig. 11A). These results strongly support the idea that a lack of the ability to escape macrophages, due to decreased host cell death, contributes to decreased proliferation of the mutant within macrophages and eventually leads to more-efficient clearance of *alg3*Δ cells.

On the other hand, to examine whether the presence of truncated core *N*-glycans lacking xylose, which are commonly generated by *ALG3*, *ALG9*, and *ALG12* mutations, could affect the immune response of host cells, we quantified the production of tumor necrosis factor alpha (TNF-α) after coculturing *cap59*Δ and *alg3*Δ *cap59*Δ strains with bone marrow-derived dendritic cells (BMDCs). We used C. neoformans *mar1*Δ *cap59*Δ, which was previously shown to activate the host cell immune response more efficiently than WT cells when cultivated in host-mimicking tissue culture medium (TC medium) (39), as a control strain. The BMDCs cocultured with the *alg3*Δ *cap59*Δ strain preincubated in TC medium showed an apparent decrease in production of TNF-α compared to the BMDCs cocultured with the *cap59*Δ and *alg3*Δ::*ALG3 cap59*Δ strains (Fig. 11B). The decreased TNF-α level in the BMDCs infected with the *alg3*Δ *cap59*Δ strain suggested that truncated core *N*-glycans are less efficient in activating host immune response than *cap59*Δ *N*-glycans. Considering that the decreased survival rate of *mar1*Δ in macrophages was partly attributable to the increased immune response of host cells (39), the results further support our speculation that the reduced survival ability of *alg3*Δ is mainly the result of decreased efficiency of host cell escape and not of the increased immune response of host cells.

### The truncated core *N*-glycans are active in inducing inflammasome-dependent inflammation of dendritic cells.

Previous studies suggested that intracellular fungal pathogens, such as C. albicans and C. neoformans, exploit macrophage pyroptosis as a host cell escape strategy that is activated by the exposure of glycosylated proteins (21, 37, 38). To examine whether the macrophage cell death, which becomes defective in the *alg3*Δ mutant, is mediated by pyroptosis, we measured the levels of interleukin-1 beta (IL-1β) secreted from the primary immune cell lines after C. neoformans infection (Fig. 11C). Since it was previously reported that phagocytosis of C. neoformans by mouse macrophages did not induce clear IL-1β production (43), we used mouse BMDCs derived from the mice deficient in ASC (apoptosis-associated speck-like protein containing a CARD) *(Asc^−^*^/^*^−^*) and gasdermin D (GSDMD) (*Gsdmd^−^*^/^*^−^*) (Fig. 11C). It was proposed that internalized fungal cells activate caspase-1 through polymerizing the cytoplasmic NLRP3 inflammasome complex that contains NLRP3, ASC, and caspase-1. Thus, *Asc^−^*^/^*^−^* mice cannot form inflammasomes, including NLRP3 inflammasomes and others. Activated caspase-1 cleaves the pro-IL-1β to mature IL-1β, as well as GSDMD, a pyroptosis executioner. GSDMD forms pores in the cell membrane to induce pyroptosis, and IL-1β is released from the cell (43). Thus, *Gsdmd^−^*^/^*^−^*cells cannot execute pyroptosis and fail to release IL-1β, although inflammasomes can be activated in the cells. We observed that IL-1β secretion was almost completely abolished in the *Asc^−^*^/^*^−^* BMDCs compared to the wild-type BMDCs upon infection by WT C. neoformans (Fig. 11C, panel a versus panel b). This indicates that C. neoformans cells induces inflammasome-dependent expression and secretion of IL-1β, strongly supporting a previous report that internalized C. neoformans activates the canonical NLRP3 inflammasome (44). Notably, in the case of *Gsdmd^−^*^/^*^−^* BMDCs (Fig. 11C, panel c), significantly decreased IL-1β levels were observed at early stages of postphagocytosis (3 h and 6 h), reflecting the release of IL-1β by pyroptosis. However, the IL-1β level was increased almost to the levels seen with the wild-type BMDCs at later stages of phagocytosis (16 h), indicating that the release of intracellular IL-1β at the later stage did not require pyroptosis but occurred mostly through nonpyroptotic host cell lysis, as observed by active lactate dehydrogenase (LDH) release (Fig. 11C), in our *in vitro* system.

On the basis of our data on the defective macrophage cell death mediated by the *alg3Δ* mutant (Fig. 7), we expected the decreased levels of IL-1β secreted from the *alg3Δ-*infected BMDCs. However, almost identical patterns of IL-1β release were observed without noticeable differences between *alg3Δ-*infected and WT C. neoformans-infected BMDCs (Fig. 11C, panels a, b, and c), indicating that the *alg3Δ* mutant was still active in inducing the inflammasome-dependent inflammation. This substantially ruled out our hypothesis that the host cell death induced by intact core *N-*glycans in C. neoformans might be mediated by pyroptosis-dependent macrophage killing mechanism as in the case of C. albicans.

## DISCUSSION

The outer glycoprotein layer of the fungal cell wall, which is highly enriched in mannose moieties, is considered a critical structure for host interactions (20). To date, only a few studies have analyzed the structure of *N*-/*O*-glycans and their roles in pathogenicity in C. neoformans, which has a capsule on the outermost cell wall layer. The *PMT* genes, which encode protein-*O*-mannosyltransferases initiating *O*-mannosylation in the ER, and *KTR3*, which encodes an α-1,2-mannosyltransferase involved in the extension of major *O*-glycans, are required for virulence in C. neoformans (32, 45). However, *OCH1*, required for the initiation of the outer mannose chain of *N*-glycans, is dispensable for virulence in C. neoformans (30), which is in stark contrast to C. albicans. Thus, in the current study, we systematically investigated the role of the core *N*-glycan structure in the pathogenicity of C. neoformans by analyzing a set of *alg* mutant strains defective in the assembly of lipid-linked *N*-glycans in the ER, thus generating truncated core *N*-glycans.

The formation of lipid-linked *N*-glycans is initiated at the cytoplasmic face of the ER membrane, where Alg7 uses UDP-GlcNAc to add GlcNAc-P to the Dol-P lipid carrier to form Dol-PP-GlcNAc, followed by sequential actions of Alg13/Alg14, Alg1, Alg2, and Alg11 on the cytoplasmic side of the ER membrane to yield Dol-PP-GlcNAc_2_Man_5_. After translocation of the heptasaccharide moiety into the ER lumen by Rft1, four more mannoses are added from the lipid-linked donor Man-P-Dol by Alg3, Alg9, and Alg12 (Fig. 1A). The resultant Dol-PP-GlcNAc_2_Man_9_ is generally extended with three glucoses from Glc-P-Dol. However, C. neoformans lacks the genes coding for Alg6, Alg8, and Alg10, which are responsible for the addition of three glucose residues (46). Thus, based on the *ALG* genes in C. neoformans, the final lipid-linked *N*-glycan synthesized in this species is expected to be GlcNAc_2_Man_9_ rather than GlcNAc_2_Man_9_Glc_3_. Finally, the multisubunit complex oligosaccharyltransferase recognizes Dol-PP-GlcNAc_2_Man_9_ as the full-length substrate and transfers the *N*-glycan moiety to asparagine residues in the Asn-X-Ser/Thr motif of proteins. The α-1,2-linked mannose residue on the middle branch of GlcNAc_2_Man_9_ assembled on proteins is expected to be removed by ER mannosidase I (47), resulting in GlcNAc_2_Man_8_ as the final mature core *N*-glycan attached to glycoproteins in the ER before transfer to the Golgi complex for further *N*-glycan modification (Fig. 1A). However, in a previous study on Dol-PP-linked glycans of C. neoformans, GlcNAc_2_Man_7_ and GlcNAc_2_Man_8_ were mainly detected, whereas GlcNAc_2_Man_9_ represented a minor fraction (46). Thus, it can be speculated that the terminal α-1,2-mannose residues of C. neoformans *N*-linked glycans might be more vulnerable to mannose trimming by ER α-1,2 mannosidases than S. cerevisiae *N*-linked glycans because of the lack of glucose residues. Consistent with that, our previous study also indicated that the mature core *N*-glycan structures assembled on cell surface mannoproteins of C. neoformans were mainly GlcNAc_2_Man_6–7_ (30), which might be generated by trimming of α-1,2-mannose residues by ER α-1,2 mannosidases.

We initially constructed the *alg3*Δ mutant, which produced truncated neutral *N*-glycans carrying five mannoses but no xylose. Notably, although moderate changes in resistance to various stressors and virulence-associated phenotypes were observed *in vitro*, the *alg3*Δ mutant was almost completely avirulent in a murine model of systemic cryptococcosis. Significantly fewer cells of the *alg3Δ* mutant were retrieved from not only the lungs but also other systemic organs such as brain, liver, and spleens of infected mice than from those of the mice infected with the WT strain or the *och1Δ* strain (see Fig. S7A in the supplemental material), indicating that pulmonary escape and dissemination of the *alg3*Δ mutant was strictly restricted. Interestingly, the lungs from the mice infected with the WT, *alg3Δ*::*ALG3*, and *och1Δ* strains exhibited large lesions with swollen granuloma and increased weight of dry tissues compared to the lungs from *alg3Δ* mutant-infected mice (Fig. S7B and C). We found that loss of Alg3 did not affect the early stages of interaction with host cells, including attachment to lung epithelial cells, opsonic/nonopsonic phagocytosis, and manipulation of phagosome acidification, but resulted in failure to trigger macrophage cell death. The capacity for cell wall remodeling after phagocytosis was retained in the *alg3*Δ mutant, strongly indicating that alteration of the core *N*-glycan structure impairs a process following cell wall remodeling after phagocytosis (highly likely the generation of a signal to host cells for induction of cell death). As observed for the *alg3*Δ cells, the *alg9*Δ and *alg12*Δ strains, which produced truncated core *N*-glycans as a major species, also showed noticeably reduced activity in inducing macrophage cell death and virulence. In clear contrast, the *och1*Δ mutant, carrying intact core *N*-glycans without outer mannose chains, induced levels of macrophage cell death comparable to those seen with the WT strain, retaining full pathogenicity. Comparison of core *N*-glycan structures generated by the mutant and WT strains strongly indicated that the upper-arm α-1,2-Man-α-1,6-Man moiety of branch c (Fig. 9E), which was absent in *N*-glycans of the *alg* mutants, might be a critical structure in promoting macrophage cell death. The host cell environment rapidly induces structural changes in the cell walls of pathogenic yeasts, affecting the exposure of pathogen-associated molecular patterns (unmasking of epitopes) as a strategy to regulate macrophage lysis. The upper-arm α-1,2-Man-α-1,6-Man disaccharide moiety on the intact core *N*-glycans of C. neoformans, which is missing in the core *N*-glycans generated by *alg* mutants, might be recognized by as-yet-undefined host macrophage receptors for induction of host cell death. Accordingly, truncated core *N*-glycans of the *alg* mutants might be inefficient in interacting with host macrophage receptors and therefore might be unable to induce macrophage host cell death.

10.1128/mBio.00711-20.8 FIG S7 Fungal burden analysis in different organs and lung morphology in the mice infected with C. neoformans. (A) Distribution of fungal cells in the systemic organs (lungs, brain, liver, and spleen) of infected mice at the humane endpoint of the experiment carried out as described in the Fig. 10C legend. Organs infected with the WT, *alg3*Δ, *alg3*Δ::*ALG3*, and *och1*Δ strains were collected, and fungal burdens were monitored in organs by determining CFU levels upon plating on YPD medium. Five mice for each strain were used for the experiments, and horizontal bars in each graph represent the average CFU counts. (B) Lung dry weight of infected mice. All the statistical analysis was performed using an unpaired two tailed Student’s *t* test (**, *P* < 0.0005; ****, *P* < 0.0001 [WT versus *alg3*Δ strain]). (C) Representative images of severe swelling and lung injury in infected mice. Download FIG S7, PDF file, 0.1 MB. Copyright © 2020 Thak et al. 2020 Thak et al. This content is distributed under the terms of the Creative Commons Attribution 4.0 International license.

**FIG 12:**
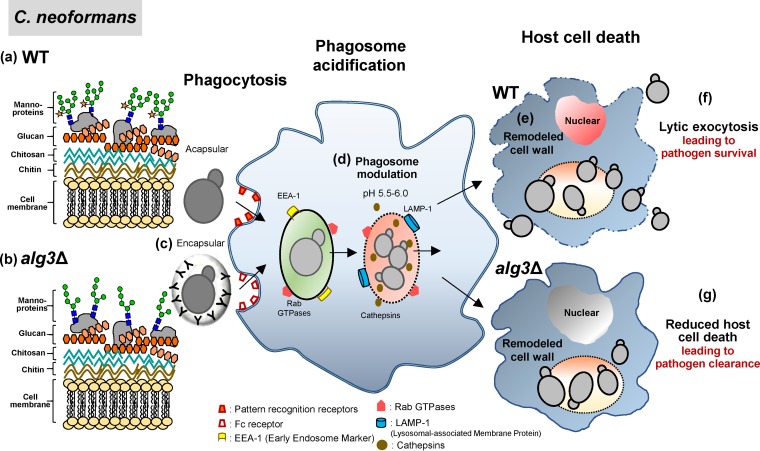
Schematic summary of the interaction of WT and *alg3*Δ C. neoformans with macrophages during host infection. (a and b) Acapsular WT C. neoformans with complete *N*-glycans (a) and the *alg3*Δ mutant strain with truncated core *N*-glycans (b) directly bind pattern recognition receptors on the macrophage cells. (c) Antibody opsonization is required for encapsular C. neoformans to be taken up by macrophages. (d) C. neoformans cells survive and replicate in the phagosome by manipulating C. neoformans-containing phagosome, leading to an increase in pH, loss of phagosomal membrane integrity, and, finally, leakage of the contents into the cytoplasm. (e) Macrophage death, characterized by phagocyte membrane lysis, is induced by C. neoformans cells, following active cell wall remodeling in the host environment. (f) Exposure of intact core *N*-glycans of C. neoformans by cell wall remodeling triggers host cell death, regardless of the presence or absence of outer mannose chains, leading to escape of C. neoformans cells from host cells for dissemination. (g) Truncated core *N*-glycans of *alg* mutants are defective in inducing host cell death, thus preventing the escape from the macrophages and leading to efficient clearance of *alg* mutant cells in the lungs.

Collectively, our results strongly indicate that the presence of structurally altered core *N*-glycans might be tolerable in the early stages of interaction with host cells during pulmonary infection, including cell wall remodeling, but might fail to induce macrophage lysis after phagocytosis to escape host cells (Fig. 12). We suggest that delayed macrophage death provides the host cells with sufficient time to produce antimicrobial mediators and combat the fungus, eventually eliminating the captured *alg* mutant cells more efficiently and thus leading to defective macrophage escape and prevention of dissemination from the lungs. Considering that the *alg9*Δ and *alg12*Δ strains were found to be more robust than the *alg3*Δ strain under most stress conditions (Fig. 10A), they might survive longer in the contained macrophage, thus generating less reduced virulence (Fig. 10B and C). The *alg3*Δ strain showed decreased levels of TNF-α production compared to WT cells, indicating that the reduced virulence of *alg* mutants is not due to the increased immune response of host cells (Fig. 11B). The absence of xylose addition as one of the modifications of the core *N*-glycans in the Golgi complex might not significantly affect C. neoformans virulence, based on a previous report that *N*-glycans of highly virulent serotype B strains do not have xylose residues (30).

Infections elicit diverse responses in the host, including activation of the innate immune system, inflammation, and cell death. The death of an infected host cell often occurs concomitantly with the death of the infecting agent, thus promoting efficient pathogen clearance. On the other hand, pathogens have evolved mechanisms to manipulate cell death for their advantage (48). Specifically, intracellular pathogens have evolved strategies to exploit host cell death as a strategy to achieve host cell escape (21). Therefore, the outcomes of host cell death, whether it is protective for the host or favorable for pathogen dissemination, are highly complex and involve a delicate balance between pro- and antideath strategies for the benefit of both the host and the pathogen. There are several types of host cell death activated by infected pathogens, including apoptosis, necroptosis, pyroptosis, and oncosis (48), and C. neoformans can damage host cells via diverse mechanisms (49). In the present report, we present a line of data supporting the idea that *N*-glycans are important in inducing lytic exocytosis, which is incongruent with a previous report that the host cell lysis by C. albicans and C. neoformans is triggered by a mechanism that is dependent on cell wall mannosylation of the cell wall (38). Although pyroptosis, a form of necrotic cell death mediated by inflammasomes, was proposed to be a specific mechanism of macrophage cell death induced by the exposed glycans assembled on glycoproteins on yeast cell wall, we were not able to prove that pyroptosis is a major mechanism of *N*-glycan-mediated host cell lysis in C. neoformans based on the release of IL-1β in *Gsdmd^−^*^/^*^−^* cells (Fig. 11C). Previous studies on the macrophages infected by C. neoformans reported that there is no pathway that predominates in the triggering of macrophage cell death, as determined by the mixed pattern of activation of death pathways (50, 51), suggesting that simultaneous occurrences of interference in multiple pathways, despite being minimal in magnitude, would result in significant effects on macrophage mortality. Thus, defining a specific host cell death pathway predominantly activated by C. neoformans *N*-glycans after phagocytosis, which requires the intact core structure of *N*-glycans, remains challenging.

Several studies have shown the importance of specific *N*-glycan structures in determining the efficiency of the interaction with proteins, such as the recognition of the upper-arm α-1,2-Man-α-1,6-Man disaccharide moiety on core *N*-glycans by Mns1 (41) and of the terminal α-1,6-Man residue by Htm1 in the ER of S. cerevisiae (52). Therefore, if *N*-glycans serve as the ligand for host receptors to trigger macrophage cell death, it is likely that the truncated core *N*-glycans generated by *alg* mutants are incapable of interacting with those receptors and thus cannot induce macrophage cell death. On the other hand, we previously reported that the third mannose in C. neoformans *O*-glycans is attached via an α-1,6 linkage instead of an α-1,2 linkage, which is distinct from what is known of most other fungal *O*-glycans (32). Thus, it is noteworthy that the C. neoformans mannotetraose *O*-glycans also carry the α-1,2-Man-α-1,6-Man moiety as a terminal structure, like the intact core *N*-glycan. It will be interesting to investigate whether a specific *O*-glycan structure is also required for macrophage cell death.

In conclusion, this study demonstrated that the structure of core *N*-glycans is critical for full virulence of C. neoformans by triggering macrophage cell death after phagocytosis. Systematic analysis of the roles of specific *N*-glycan structures in the interaction with macrophage receptors or the binding to signaling materials might elucidate the mechanism by which mannoproteins at the cell surface are involved in triggering macrophage cell death, which appears to be mediated by an as-yet-unidentified pathway, to escape host immunity. In particular, in C. neoformans covered with the capsule, which might block access to the cell wall and membrane, it is challenging to elucidate how signals generated by cell surface-associated molecular components can be recognized by host cells. Study of cell wall remodeling in fungal pathogenesis at the molecular level, focusing on the roles of *N*-/*O*-glycan structures in the interaction with host cells, will provide deep insight into the mechanism of fungal infection, which would be useful for the development of novel antifungal agents targeting *N*-/*O*-glycan structures.

## MATERIALS AND METHODS

### Strains, media, plasmids, and primers.

The C. neoformans strains used in this study are listed in Table S1A in the supplemental material. Yeast cells were generally cultured in YPD medium (1% [wt/vol] yeast extract, 2% [wt/vol] peptone, 2% [wt/vol] dextrose) with shaking (220 rpm) at 30°C. Yeast cells were cultured in Synthetic Complete (SC) medium (0.67% yeast nitrogen base without amino acids, 2% glucose, and complete supplement mixture of amino acids) for analysis of secreted proteins. The plasmids and primers used in this study are listed in Table S1B and C, respectively. Construction of the C. neoformans acapsular *cap59*Δ strain, *alg* mutant (*alg3*Δ, *alg9*Δ, and *alg12*Δ) strains, and recombinant C. neoformans strains expressing His-tagged Plb1 and MP98 is described in Text S1 in the supplemental material. C. neoformans transformants were selected on YPD solid medium containing 100 μg/ml nourseothricin acetyltransferase (Jena Bioscience), referred to here as YPD_NAT_, or on YPD solid medium containing 200 μg/ml G418 disulfate (Duchefa), referred to here as YPD_G418_, or on YPD solid medium containing 100 μg/ml hygromycin B (Sigma), referred to here as YPD_HYB_. For cultivation under host-mimicking tissue culture conditions, C. neoformans cells were incubated in CO_2_-independent tissue culture medium (TC medium; Gibco) with shaking at 37°C. Gene sequence information was obtained from the C. neoformans serotype A genome database (Duke University/Broad Institute of Harvard and NCBI).

10.1128/mBio.00711-20.1 TEXT S1 Supplemental methods and references. Download Text S1, DOCX file, 0.02 MB. Copyright © 2020 Thak et al. 2020 Thak et al. This content is distributed under the terms of the Creative Commons Attribution 4.0 International license.

10.1128/mBio.00711-20.9 TABLE S1 (A) Strains used in this study. (B) Plasmids used in this study. (C) Oligonucleotides used in this study. Download Table S1, DOCX file, 0.02 MB. Copyright © 2020 Thak et al. 2020 Thak et al. This content is distributed under the terms of the Creative Commons Attribution 4.0 International license.

### HPLC and MALDI-TOF analysis of *N*-linked glycans from cell wall mannoproteins.

cwMPs were isolated from C. neoformans cells as described previously (30, 53). *N*-linked glycans were released from the purified cwMPs using PNGase F (New England Biolabs) and were purified over a Carbograph Extract-Clean (Grace) column. The *N*-glycans were labeled with 2-aminobenzoic acid (2-AA; Sigma) and purified using a Cyano Base cartridge (Bond Elut-CN-E; Agilent) (100 mg) to remove excess 2-AA. Purified *N*-glycans were reacted with α-1,2 mannosidase (α-1,2 MNS; Prozyme) and subsequently with α-1,6 mannosidase (α-1,6 MNS; New England Biolabs). To remove enzymes, the *N*-glycans were purified using a 30K Microcon device (Millipore). 2-AA-labeled oligosaccharides were analyzed with a Waters 2690 HPLC system and a 2475 fluorescence detector with excitation and emission wavelengths of 360 nm and 425 nm, respectively. Data were collected using Empower 2 software (Waters). For MALDI-TOF analysis, the matrix solution was prepared as previously described (53) and was mixed with samples of equal volume. Mixed glycan samples were spotted on a MSP 96 polished-steel target (Bruker Daltonics). Crystallized samples were analyzed using a Microflex mass spectrometer (Bruker Daltonics).

### *In vitro* virulence-associated factor production assay.

For the induction of capsule formation, C. neoformans cells were grown on liquid Sabouraud dextrose medium (Difco) at 30°C for 16 h and then incubated in 10% Sabouraud dextrose medium (pH 7.3) with 50 mM morpholinepropanesulfonic acid (MOPS) at 30°C for 2 days. A Zeiss Axioscope (A1) equipped with an AxioCam MRm digital camera was used to visualize India ink-stained C. neoformans cells. Capsule and yeast cell body diameters were measured using ImageJ (National Institutes of Health). To examine melanin production, C. neoformans cells were grown overnight at 30°C in YPD medium, diluted in phosphate-buffered saline (PBS) to an optical density at 600 nm of 0.6, spotted onto a l-DOPA plate (7.6 mM l-asparagine monohydrate, 5.6 mM glucose, 22 mM KH_2_PO_4_, 1 mM MgSO_4_·7H_2_O, 0.5 mM l-DOPA, 0.3 mM thiamine-HCl, and 20 nM biotin), and incubated at 30°C and 37°C for 2 days.

### Animal study.

Animal studies were conducted at the Chung-Ang University Animal Experiment Center and were approved by the Ministry of Food and Drug Safety (MFDS, South Korea). The experimenter completed an animal experimental education and followed the guidelines. Survival and fungal burden were assayed as described previously (54). Briefly, 10 mice (6-week-old female A/J Slc mice; Japan SLC) per strain were infected via intranasal instillation with 10^5^ cells. Mice were weighed and monitored once daily and sacrificed when they lost 30% of original body weight rapidly or exhibited signs of morbidity. Kaplan-Meier survival curves were generated using Prism version 5.02 (GraphPad Software). For the fungal burden assay, the lungs of C. neoformans-infected mice (*n* = 5 per strain) were dissected on day 7. Lungs were excised, and half-organ portions were homogenized, serially diluted, and plated onto YPD medium containing 100 μg/ml chloramphenicol (Sigma). For histopathological analysis, fixed half-lung samples were sectioned and stained with mucicarmine (Abcam). C. neoformans colonization was analyzed by microscopy. Images were captured using a Zeiss Axioscope (A1) equipped with an AxioCam MRm digital camera.

### *In vitro* host cell interaction analysis.

To analyze the adhesion of yeast cells to (A549) epithelial lung cells, 2 × 10^5^ A549 cells were seeded into 24-well plates in Dulbecco’s modified Eagle’s medium (DMEM; Gibco) supplemented with 10% fetal bovine serum (FBS) and were cultivated at 37°C in 5% CO_2_ for 18 h. C. neoformans cells (2 × 10^6^) were added to each well (C. neoformans/A549 ratio, 10:1) and coincubated at 37°C for 1 h. Then, each well was gently washed three times with PBS to remove nonadherent yeast cells. The A549 cells were lysed with sterile cold water. The lysates were spread on YPD plates, and CFU counting was performed to determine viability. For the macrophage phagocytosis assay, 1 × 10^5^ J774A.1 macrophages were seeded into 24-well plates in Dulbecco’s modified Eagle’s medium (DMEM; Gibco) supplemented with 10% fetal bovine serum (FBS) and cultivated at 37°C in 5% CO_2_ for 18 h. The J774A.1 macrophages were activated by adding 150 ng/ml phorbol 12-myristate 13-acetate (PMA; Sigma) into serum-free DMEM for 1 h. Encapsular yeast cells were opsonized with 10 μg/ml mouse immunoglobulin G 18B7 antibody (kindly gifted by Arturo Casadevall, Johns Hopkins School of Public Health) at 37°C for 1 h. C. neoformans cells (1 × 10^6^) were added to each well (C. neoformans/J774A.1 ratio, 10:1) and incubated with serum-free DMEM. After 2 h of coincubation, nonphagocytized yeast cells were removed by washing three times with PBS, and the macrophages were lysed in distilled water for 30 min. Viability was assessed as described for CFU analysis in three independent experiments.

### Phagosomal maturation assessment.

J774A.1 cells (1 × 10^5^) were seeded onto 12-mm-diameter Flux film coverslips (SPL Life Sciences) in the wells of a 24-well plate and activated as described above. Encapsular and acapsular C. neoformans cells were heat-killed at 56°C for 30 min to serve as a positive control for phagosomal maturation. J774A.1 cells were infected with 1 × 10^6^ yeast cells with or without opsonization at a multiplicity of infection (MOI) of 10:1. At the same time, wells were replenished with serum-free DMEM containing 50 nM LysoTracker Red DND-99 (Invitrogen) for fluorescence detection of acidic cellular compartments. At 2 h postincubation, the cells were fixed in 3.7% formaldehyde and coverslips were mounted onto glass slides and observed under a Zeiss confocal microscope using excitation and emission wavelengths of 555 nm and 573 nm, respectively. The numbers of matured phagosomes were determined by counting LysoTracker Red-stained phagolysosomes with a 650 detector gain value among cryptococcus-containing phagosomes. The percentage of acidified macrophages among cryptococcus-containing phagolysosomes was calculated by analyzing at least 100 infected macrophages per infection in three independent experiments.

### Quantification of macrophage death by C. neoformans.

J774A.1 cells (5 × 10^5^) were seeded onto 12-mm-diameter Flux film coverslips in the wells of a 24-well plate, cultivated in RPMI 1640 medium (HyClone) containing 3% FBS, and incubated at 37°C in 5% CO_2_ for 18 h. C. neoformans cells maintained with or without opsonization were diluted to 1 × 10^6^ cells/ml in RPMI 1640 medium and added to the activated macrophages for 1 h. After 1 h of coincubation, nonphagocytized C. neoformans cells were removed by washing with PBS and the medium was replaced with RPMI medium containing 1 μg/ml propidium iodide (PI; Sigma). After incubation for the indicated times (4 h, 8 h, and 12 h), the cells were fixed in 3.7% formaldehyde and coverslips were mounted onto glass slides and observed under a Zeiss confocal microscope at an excitation wavelength of 555 nm and an emission wavelength of 573 nm. The percentages of PI-stained dead macrophages determined with a 650 detector gain value were calculated among at least 100 infected macrophages per infection with three independent experiments.

Macrophage cell death, induced by cell wall remodeling of C. neoformans cells after phagocytosis, was analyzed as previously reported (38). Briefly, J774A.1 cells (1 × 10^5^/ml) were seeded into 6-well plates containing RPMI 1640 medium with 3% FBS and were incubated at 37°C in 5% CO_2_. C. neoformans cells (1 × 10^6^/ml) were added to the activated macrophages and coincubated for 4 h. The nonphagocytized cells were collected by centrifugation of the culture supernatant, and the phagocytized cells were separately collected by lysing the macrophages. Both the nonphagocytized and phagocytized C. neoformans cells were heat-killed at 56°C for 30 min, stained with calcofluor white (CFW), and used for subsequent infection with activated macrophages for 1 h. After washing with PBS were performed three times, the infected macrophages were further incubated with RPMI medium containing 1 μg/ml PI for 4 h, and the CFW-stained C. neoformans cells present in macrophages were observed by confocal microscopy at excitation 380 nm/emission 475 nm.

### Staining of chitin and chitosan in C. neoformans cells.

To stain chitin, C. neoformans cells were washed in PBS and incubated with 100 μg/ml fluorescein isothiocyanate (FITC)-conjugated wheat germ agglutinin (WGA-FITC; Invitrogen) for 1 h. The stained cells were washed with PBS three times and resuspended in PBS. The chitin stained with WGA-FITC in the cell wall was observed by Zeiss confocal microscope (excitation 480 nm, emission 535 nm). To visualize chitosan, cells were washed with McIlvaine’s buffer (0.2 M Na_2_HPO_4_, 0.1 M citric acid, pH 6.0) and stained with 300 μg/ml eosin Y (Sigma) in McIlvaine’s buffer for 30 min. The stained cells were washed with McIlvaine’s buffer three times and resuspended in McIlvaine’s buffer. The chitosan stained with eosin Y in the cell wall was observed by the use of a Zeiss confocal microscope (excitation 534 nm, emission 544 nm). The mean intensity of cells stained with WGA-FITC and eosin Y was measured using NIS-Elements image analysis software (Nikon). The microscopy images, which overlapped the confocal images, were obtained by differential interference contrast (DIC).

### Generation of bone marrow-derived macrophages and dendritic cells.

Bone marrow progenitor cells were isolated from femurs of C57BL/6J mice (The Jackson Laboratory), *Asc*-deficient mice (MTA between Genentech and ML Shinohara), and *Gsdmd*-deficient mice (a gift from RE Vance at the University of California, Berkeley). Briefly, femurs and tibias isolated from mice were cut at both ends, and each bone was flushed with 5 to 10 ml cold PBS using a 27-gauge needle. Red blood cells were lysed in 1× RBC lysis buffer (0.83% ammonium chloride). Bone marrow progenitor cells were resuspended in BMDM medium (1× DMEM with 4.5 g/liter d-glucose and l-glutamine and 110 mg/liter sodium pyruvate, 10% FBS, 1 U/ml penicillin/streptomycin) or BMDC medium (1× RPMI medium, 10% FBS, 1 U/ml penicillin/streptomycin). To obtain the differentiated bone marrow macrophage cells (BMDMs), bone marrow progenitor cells were incubated in BMDM medium with 3 ng/ml recombinant mouse granulocyte-macrophage colony-stimulating factor (GM-CSF) (rmGM-CSF; R&D Systems or BioLegend) at 37°C with 5% CO_2_. The medium was refreshed after 3 to 4 days, and the cells were harvested on day 7. Activated bone marrow dendritic cells (BMDCs) were obtained in BMDC medium in the presence of 20 ng/ml rmGM-CSF, and additional medium was added after 3 days. On day 7, floating BMDCs were harvested by centrifugation.

### Fungal cell survival analysis in BMDMs.

BMDMs (1 × 10^5^) were seeded into 96-well plates in DMEM medium (Gibco) containing 10% FBS and incubated at 37°C in 5% CO_2_ for 18 h. BMDMs were incubated with fungal cells of the encapsular and acapsular background strains at an MOI of 1 for 1 h at 37°C with 5% CO_2_. Each well was washed 3 times with 200 μl PBS to remove extracellular yeast cells. After addition of 100 μl macrophage media to each well, the C. neoformans-infected BMDMs were incubated for 24 h. Then, BMDMs were lysed in distilled water with pipetting of the contents up and down several times, and the phagocytosed fungal cells were collected, serially diluted, and plated onto a YPD plate to assess the number of viable C. neoformans cells.

### Cytokine ELISA and LDH analysis.

Activated BMDCs were seeded in 96-well plates at 1 × 10^5^ cells/well (in RPMI media plus heat-inactivated FBS 10%) and incubated at 37°C in 5% CO_2_ for 1 h. C. neoformans cells were washed twice with PBS, counted, and added to BMDCs at a concentration of 1 × 10^6^ cells/well. After the indicated time (3 h, 6 h, or 16 h), supernatants were collected and centrifuged at 17,000 × *g* for 5 min. Levels of released tumor necrosis factor alpha (TNF-α) and interleukin-1β (IL-1β) were analyzed by enzyme-linked immunosorbent assay (ELISA) using mouse TNF-α and IL-1β ELISA Max set deluxe kits (BioLegend), according to the manufacturer’s instructions. Levels of lactate dehydrogenase (LDH) enzyme released into the cell culture supernatant were measured as a colorimetric change using an LDH cytotoxicity detection kit (Pierce) according to the manufacturer’s protocol. The plate was read at 490 nm using a microplate reader (Tecan) after 30 min incubation at room temperature. The absorbance was measured, and percent cytotoxicity was calculated for the samples.

## References

[1] Helenius A, Aebi M 2004 Roles of *N*-linked glycans in the endoplasmic reticulum. Annu Rev Biochem 73:1019–1049. doi:10.1146/annurev.biochem.73.011303.073752. 15189166

[2] Varki A, Gagneux P, 2015 Biological functions of glycans, p 77–88. *In* Varki A, Cummings RD, Esko JD, Stanley P, Hart GW, Aebi M, Darvill AG, Kinoshita T, Packer NH, Prestegard JH, Schnaar RL, Seeberger PH (ed), Essentials of glycobiology. Cold Spring Harbor Laboratory Press, Cold Spring Harbor, NY. doi:10.1093/glycob/cwv091. 28876862

[3] Bates S, Hughes HB, Munro CA, Thomas WP, MacCallum DM, Bertram G, Atrih A, Ferguson MA, Brown AJ, Odds FC, Gow NA 2006 Outer chain *N*-glycans are required for cell wall integrity and virulence of *Candida albicans*. J Biol Chem 281:90–98. doi:10.1074/jbc.M510360200. 16263704

[4] Bates S, Hall RA, Cheetham J, Netea MG, MacCallum DM, Brown AJ, Odds FC, Gow NA 2013 Role of the *Candida albicans* MNN1 gene family in cell wall structure and virulence. BMC Res Notes 6:294. doi:10.1186/1756-0500-6-294. 23886038 PMC3750861

[5] Hall RA, Bates S, Lenardon MD, Maccallum DM, Wagener J, Lowman DW, Kruppa MD, Williams DL, Odds FC, Brown AJ, Gow NA 2013 The MnnII mannosyltransferase family modulates mannoprotein fibril length, immune recognition and virulence of *Candida albicans*. PLoS Pathog 9:e1003276. doi:10.1371/journal.ppat.1003276. 23633946 PMC3636026

[6] Mora-Montes HM, Bates S, Netea MG, Castillo L, Brand A, Buurman ET, Diaz-Jimenez DF, Jan Kullberg B, Brown AJ, Odds FC, Gow NA 2010 A multifunctional mannosyltransferase family in *Candida albicans* determines cell wall mannan structure and host-fungus interactions. J Biol Chem 285:12087–12095. doi:10.1074/jbc.M109.081513. 20164191 PMC2852947

[7] Teixeira PA, Penha LL, Mendonca-Previato L, Previato JO 2014 Mannoprotein MP84 mediates the adhesion of *Cryptococcus neoformans* to epithelial lung cells. Front Cell Infect Microbiol 4:106. doi:10.3389/fcimb.2014.00106. 25191644 PMC4137752

[8] Munro S 2001 What can yeast tell us about *N*-linked glycosylation in the Golgi apparatus? FEBS Lett 498:223–227. doi:10.1016/s0014-5793(01)02488-7. 11412862

[9] Lehle L, Strahl S, Tanner W 2006 Protein glycosylation, conserved from yeast to man: a model organism helps elucidate congenital human diseases. Angew Chem Int Ed Engl 45:6802–6818. doi:10.1002/anie.200601645. 17024709

[10] Gemmill TR, Trimble RB 1999 Overview of *N*- and *O*-linked oligosaccharide structures found in various yeast species. Biochim Biophys Acta 1426:227–237. doi:10.1016/S0304-4165(98)00126-3. 9878752

[11] Bai C, Xu XL, Chan FY, Lee RT, Wang Y 2006 MNN5 encodes an iron-regulated alpha-1,2-mannosyltransferase important for protein glycosylation, cell wall integrity, morphogenesis, and virulence in *Candida albicans*. Eukaryot Cell 5:238–247. doi:10.1128/EC.5.2.238-247.2006. 16467465 PMC1405895

[12] Zhang SQ, Zou Z, Shen H, Shen SS, Miao Q, Huang X, Liu W, Li LP, Chen SM, Yan L, Zhang JD, Zhao JJ, Xu GT, An MM, Jiang YY 2016 Mnn10 maintains pathogenicity in *Candida albicans* by extending alpha-1, 6-mannose backbone to evade host dectin-1 mediated antifungal immunity. PLoS Pathog 12:e1005617. doi:10.1371/journal.ppat.1005617. 27144456 PMC4856274

[13] West L, Lowman DW, Mora-Montes HM, Grubb S, Murdoch C, Thornhill MH, Gow NA, Williams D, Haynes K 2013 Differential virulence of *Candida glabrata* glycosylation mutants. J Biol Chem 288:22006–22018. doi:10.1074/jbc.M113.478743. 23720756 PMC3724654

[14] Pérez-García LA, Csonka K, Flores-Carreón A, Estrada-Mata E, Mellado-Mojica E, Németh T, López-Ramírez LA, Toth R, López MG, Vizler C, Marton A, Tóth A, Nosanchuk JD, Gácser A, Mora-Montes HM 2016 Role of protein glycosylation in *Candida parapsilosis* cell wall integrity and host interaction. Front Microbiol 7:306. doi:10.3389/fmicb.2016.00306. 27014229 PMC4781877

[15] Garfoot AL, Goughenour KD, Wuthrich M, Rajaram MVS, Schlesinger LS, Klein BS, Rappleye CA 2018 *O*-Mannosylation of proteins enables Histoplasma yeast survival at mammalian body temperatures. mBio 9:e02121-17. doi:10.1128/mBio.02121-17. 29295913 PMC5750402

[16] Kotz A, Wagener J, Engel J, Routier FH, Echtenacher B, Jacobsen I, Heesemann J, Ebel F 2010 Approaching the secrets of *N*-glycosylation in *Aspergillus fumigatus*: characterization of the AfOch1 protein. PLoS One 5:e15729. doi:10.1371/journal.pone.0015729. 21206755 PMC3012087

[17] Rajasingham R, Smith RM, Park BJ, Jarvis JN, Govender NP, Chiller TM, Denning DW, Loyse A, Boulware DR 2017 Global burden of disease of HIV-associated cryptococcal meningitis: an updated analysis. Lancet Infect Dis 17:873–881. doi:10.1016/S1473-3099(17)30243-8. 28483415 PMC5818156

[18] Sabiiti W, May RC 2012 Mechanisms of infection by the human fungal pathogen *Cryptococcus neoformans*. Future Microbiol 7:1297–1313. doi:10.2217/fmb.12.102. 23075448

[19] Erwig LP, Gow NA 2016 Interactions of fungal pathogens with phagocytes. Nat Rev Microbiol 14:163–176. doi:10.1038/nrmicro.2015.21. 26853116

[20] Gow NAR, Latge JP, Munro CA 2017 The fungal cell wall: structure, biosynthesis, and function. Microbiol Spectr 5(3). doi:10.1128/microbiolspec.FUNK-0035-2016. PMC11687499 28513415

[21] May RC, Casadevall A 2018 In fungal intracellular pathogenesis, form determines fate. mBio 9:e02092-18. doi:10.1128/mBio.02092-18. 30352939 PMC6199499

[22] De Leon-Rodriguez CM, Rossi DCP, Fu MS, Dragotakes Q, Coelho C, Guerrero Ros I, Caballero B, Nolan SJ, Casadevall A 2018 The outcome of the *Cryptococcus neoformans*-macrophage interaction depends on phagolysosomal membrane integrity. J Immunol 201:583–603. doi:10.4049/jimmunol.1700958. 29858266 PMC6245949

[23] Denham ST, Brown J 17 2 2018, posting date Mechanisms of pulmonary escape and dissemination by *Cryptococcus neoformans*. J Fungi (Basel) doi:10.3390/jof4010025. PMC5872328 29463005

[24] Cox GM, McDade HC, Chen SC, Tucker SC, Gottfredsson M, Wright LC, Sorrell TC, Leidich SD, Casadevall A, Ghannoum MA, Perfect JR 2001 Extracellular phospholipase activity is a virulence factor for *Cryptococcus neoformans*. Mol Microbiol 39:166–175. doi:10.1046/j.1365-2958.2001.02236.x. 11123698

[25] Fu MS, Coelho C, De Leon-Rodriguez CM, Rossi DCP, Camacho E, Jung EH, Kulkarni M, Casadevall A 2018 *Cryptococcus neoformans* urease affects the outcome of intracellular pathogenesis by modulating phagolysosomal pH. PLoS Pathog 14:e1007144. doi:10.1371/journal.ppat.1007144. 29906292 PMC6021110

[26] Kronstad JW, Attarian R, Cadieux B, Choi J, D'Souza CA, Griffiths EJ, Geddes JMH, Hu G, Jung WH, Kretschmer M, Saikia S, Wang J 2011 Expanding fungal pathogenesis: *Cryptococcus* breaks out of the opportunistic box. Nat Rev Microbiol 9:193–203. doi:10.1038/nrmicro2522. 21326274 PMC4698337

[27] Lin X, Heitman J 2006 The biology of the *Cryptococcus neoformans* species complex. Annu Rev Microbiol 60:69–105. doi:10.1146/annurev.micro.60.080805.142102. 16704346

[28] O'Meara TR, Alspaugh JA 2012 The *Cryptococcus neoformans* capsule: a sword and a shield. Clin Microbiol Rev 25:387–408. doi:10.1128/CMR.00001-12. 22763631 PMC3416491

[29] Cadieux B, Lian T, Hu G, Wang J, Biondo C, Teti G, Liu V, Murphy ME, Creagh AL, Kronstad JW 2013 The Mannoprotein Cig1 supports iron acquisition from heme and virulence in the pathogenic fungus *Cryptococcus neoformans*. J Infect Dis 207:1339–1347. doi:10.1093/infdis/jit029. 23322859 PMC3603535

[30] Park JN, Lee DJ, Kwon O, Oh DB, Bahn YS, Kang HA 2012 Unraveling unique structure and biosynthesis pathway of *N*-linked glycans in human fungal pathogen *Cryptococcus neoformans* by glycomics analysis. J Biol Chem 287:19501–19515. doi:10.1074/jbc.M112.354209. 22500028 PMC3365987

[31] Breitling J, Aebi M 2013 *N*-linked protein glycosylation in the endoplasmic reticulum. Cold Spring Harb Perspect Biol 5:a013359. doi:10.1101/cshperspect.a013359. 23751184 PMC3721281

[32] Lee DJ, Bahn YS, Kim HJ, Chung SY, Kang HA 2015 Unraveling the novel structure and biosynthetic pathway of *O*-linked glycans in the Golgi apparatus of the human pathogenic yeast *Cryptococcus neoformans*. J Biol Chem 290:1861–1873. doi:10.1074/jbc.M114.607705. 25477510 PMC4340427

[33] Garcia-Rivera J, Chang YC, Kwon-Chung KJ, Casadevall A 2004 *Cryptococcus neoformans* CAP59 (or Cap59p) is involved in the extracellular trafficking of capsular glucuronoxylomannan. Eukaryot Cell 3:385–392. doi:10.1128/ec.3.2.385-392.2004. 15075268 PMC387637

[34] Esher SK, Granek JA, Alspaugh JA 2015 Rapid mapping of insertional mutations to probe cell wall regulation in *Cryptococcus neoformans*. Fungal Genet Biol 82:9–21. doi:10.1016/j.fgb.2015.06.003. 26112692 PMC4693612

[35] DeLeon-Rodriguez CM, Casadevall A 2016 *Cryptococcus neoformans*: tripping on acid in the phagolysosome. Front Microbiol 7:164. doi:10.3389/fmicb.2016.00164. 26925039 PMC4756110

[36] Smith LM, Dixon EF, May RC 2015 The fungal pathogen *Cryptococcus neoformans* manipulates macrophage phagosome maturation. Cell Microbiol 17:702–713. doi:10.1111/cmi.12394. 25394938

[37] Krysan DJ, Sutterwala FS, Wellington M 2014 Catching fire: *Candida albicans*, macrophages, and pyroptosis. PLoS Pathog 10:e1004139. doi:10.1371/journal.ppat.1004139. 24967821 PMC4072798

[38] O'Meara TR, Veri AO, Ketela T, Jiang B, Roemer T, Cowen LE 2015 Global analysis of fungal morphology exposes mechanisms of host cell escape. Nat Commun 6:6741. doi:10.1038/ncomms7741. 25824284 PMC4382923

[39] Esher SK, Ost KS, Kohlbrenner MA, Pianalto KM, Telzrow CL, Campuzano A, Nichols CB, Munro C, Wormley FL Jr, Alspaugh JA 2018 Defects in intracellular trafficking of fungal cell wall synthases lead to aberrant host immune recognition. PLoS Pathog 14:e1007126. doi:10.1371/journal.ppat.1007126. 29864141 PMC6002136

[40] Latge JP 2017 Immune evasion: face changing in the fungal opera. Nat Microbiol 2:16266. doi:10.1038/nmicrobiol.2016.266. 28120932

[41] Cipollo JF, Trimble RB 2002 The *Saccharomyces cerevisiae* alg12delta mutant reveals a role for the middle-arm alpha1,2Man- and upper-arm alpha1,2Manalpha1,6Man- residues of Glc3Man9GlcNAc2-PP-Dol in regulating glycoprotein glycan processing in the endoplasmic reticulum and Golgi apparatus. Glycobiology 12:749–762. doi:10.1093/glycob/cwf082. 12460943

[42] Frank CG, Aebi M 2005 ALG9 mannosyltransferase is involved in two different steps of lipid-linked oligosaccharide biosynthesis. Glycobiology 15:1156–1163. doi:10.1093/glycob/cwj002. 15987956

[43] Shi J, Gao W, Shao F 2017 Pyroptosis: gasdermin-mediated programmed necrotic cell death. Trends Biochem Sci 42:245–254. doi:10.1016/j.tibs.2016.10.004. 27932073

[44] Chen M, Xing Y, Lu A, Fang W, Sun B, Chen C, Liao W, Meng G 2015 Internalized *Cryptococcus neoformans* activates the canonical caspase-1 and the noncanonical caspase-8 inflammasomes. J Immunol 195:4962–4972. doi:10.4049/jimmunol.1500865. 26466953

[45] Willger SD, Ernst JF, Alspaugh JA, Lengeler KB 2009 Characterization of the PMT gene family in *Cryptococcus neoformans*. PLoS One 4:e6321. doi:10.1371/journal.pone.0006321. 19633715 PMC2711527

[46] Samuelson J, Banerjee S, Magnelli P, Cui J, Kelleher DJ, Gilmore R, Robbins PW 2005 The diversity of dolichol-linked precursors to Asn-linked glycans likely results from secondary loss of sets of glycosyltransferases. Proc Natl Acad Sci U S A 102:1548–1553. doi:10.1073/pnas.0409460102. 15665075 PMC545090

[47] Camirand A, Heysen A, Grondin B, Herscovics A 1991 Glycoprotein biosynthesis in *Saccharomyces cerevisiae*. Isolation and characterization of the gene encoding a specific processing alpha-mannosidase. J Biol Chem 266:15120–15127. 1714453

[48] Labbé K, Saleh M 2008 Cell death in the host response to infection. Cell Death Differ 15:1339–1349. doi:10.1038/cdd.2008.91. 18566602

[49] Casadevall A, Coelho C, Alanio A 2018 Mechanisms of *Cryptococcus neoformans*-mediated host damage. Front Immunol 9:855. doi:10.3389/fimmu.2018.00855. 29760698 PMC5936990

[50] Ben-Abdallah M, Sturny-Leclère A, Avé P, Louise A, Moyrand F, Weih F, Janbon G, Mémet S 2012 Fungal-induced cell cycle impairment, chromosome instability and apoptosis via differential activation of NF-kappaB. PLoS Pathog 8:e1002555. doi:10.1371/journal.ppat.1002555. 22396644 PMC3291658

[51] Coelho C, Souza ACO, Derengowski LDS, de Leon-Rodriguez C, Wang B, Leon-Rivera R, Bocca AL, Gonçalves T, Casadevall A 2015 Macrophage mitochondrial and stress response to ingestion of *Cryptococcus neoformans*. J Immunol 194:2345–2357. doi:10.4049/jimmunol.1402350. 25646306 PMC4340727

[52] Clerc S, Hirsch C, Oggier DM, Deprez P, Jakob C, Sommer T, Aebi M 2009 Htm1 protein generates the *N*-glycan signal for glycoprotein degradation in the endoplasmic reticulum. J Cell Biol 184:159–172. doi:10.1083/jcb.200809198. 19124653 PMC2615083

[53] Thak EJ, Kim J, Lee DJ, Kim JY, Kang HA 2018 Structural analysis of *N*-/*O*-glycans assembled on proteins in yeasts. J Microbiol 56:11–2354. doi:10.1007/s12275-018-7468-x. 29299842

[54] Cheon SA, Jung KW, Chen YL, Heitman J, Bahn YS, Kang HA 2011 Unique evolution of the UPR pathway with a novel bZIP transcription factor, Hxl1, for controlling pathogenicity of *Cryptococcus neoformans*. PLoS Pathog 7:e1002177. doi:10.1371/journal.ppat.1002177. 21852949 PMC3154848

