# Supplementary material for: Core N-Glycan Structures Are Critical for the Pathogenicity of Cryptococcus neoformans by Modulating Host Cell Death
Source: mBio. 2020 May 12;11(3):e00711-20. doi: 10.1128/mBio.00711-20 (PMC7218283; doi:10.1128/mBio.00711-20)
Supplement: FIG S1 [file mBio.00711-20-sf001.pdf]

Supplementary Fig. 1

A.

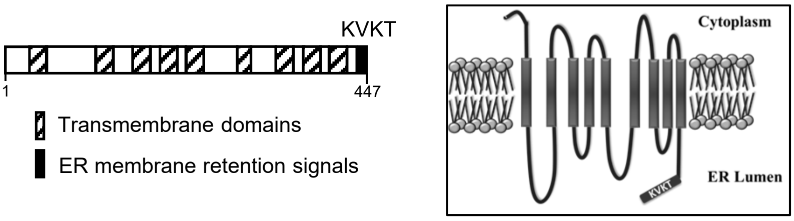

B.

Percent Identity (%)

Divergence

	CnAlg3p	ScAlg3p	HpAlg3p	CaAlg3p	AnAlg3p	SpAlg3p
**CnAlg3p**		38.3	38.6	25.1	45.5	44.5
**ScAlg3p**	145.5		43.1	33.0	40.8	39.8
**HpAlg3p**	134.1	121.8		39.0	40.5	41.4
**CaAlg3p**	156.9	121.9	106.7		26.9	27.1
**AnAlg3p**	121.1	131.0	130.2	148.1		52.6
**SpAlg3p**	130.0	144.0	129.0	152.9	114.2	

C.

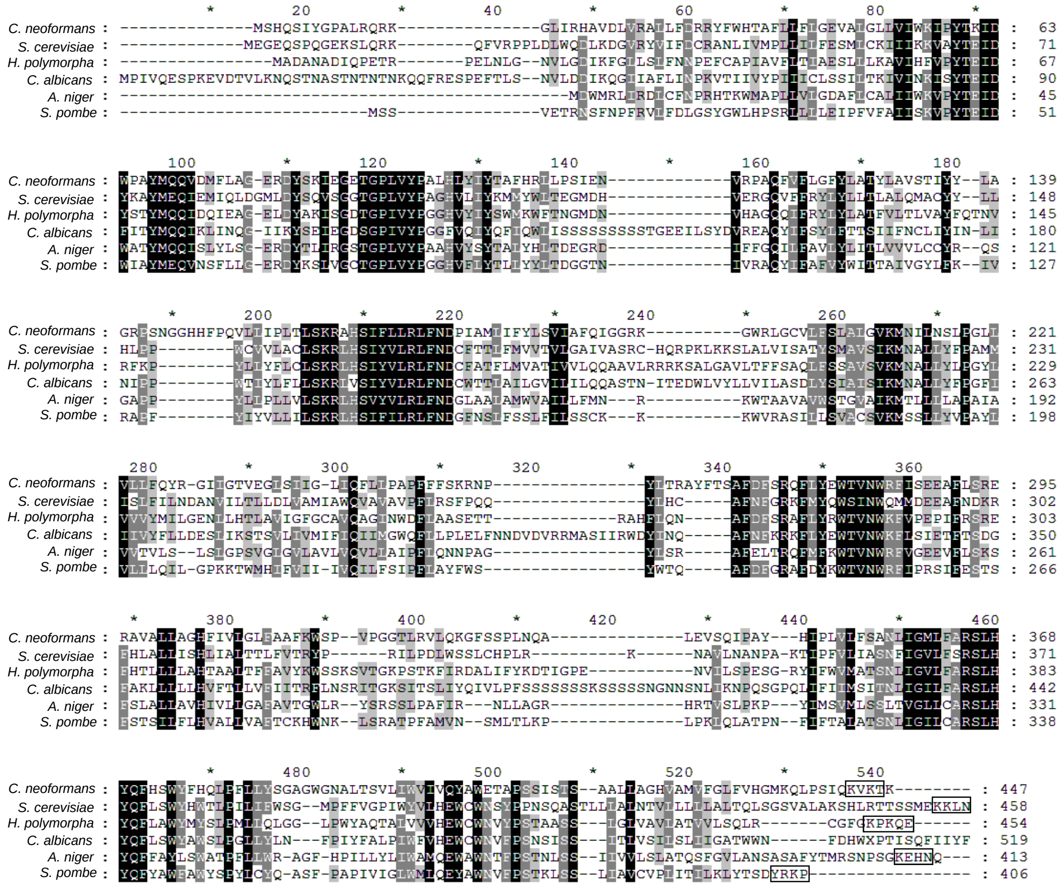

FIG S1 Bioinformatics analysis of Alg3 protein. (A) Domain structure of *C. neoformans* Alg3. Predicted transmembrane domains and ER retention signal of Alg3 are indicated by slashes and a box, respectively. (B) Sequence identities and similarities between Alg3 homologs of *S. cerevisiae* (ScAlg3: NP_009471), *H. polymorpha* (HpAlg3: ABB04524), *C. albicans* (CaAlg3: XP_712105), *A. niger* (AnAlg3: XP_001398696), and *S. pombe* (SpAlg3: NP_593853). (C) Multiple sequence alignment of Alg3 homologs generated using ClustalW 1.8. Identical residues and conservative amino acid substitutions are indicated by black and gray shading, respectively. The ER retention signal, KKXX-like (X = any amino acid) motif, is boxed.
