# Supplementary material for: Core N-Glycan Structures Are Critical for the Pathogenicity of Cryptococcus neoformans by Modulating Host Cell Death
Source: mBio. 2020 May 12;11(3):e00711-20. doi: 10.1128/mBio.00711-20 (PMC7218283; doi:10.1128/mBio.00711-20)
Supplement: FIG S2 [file mBio.00711-20-sf002.pdf]

Supplementary Fig. 2

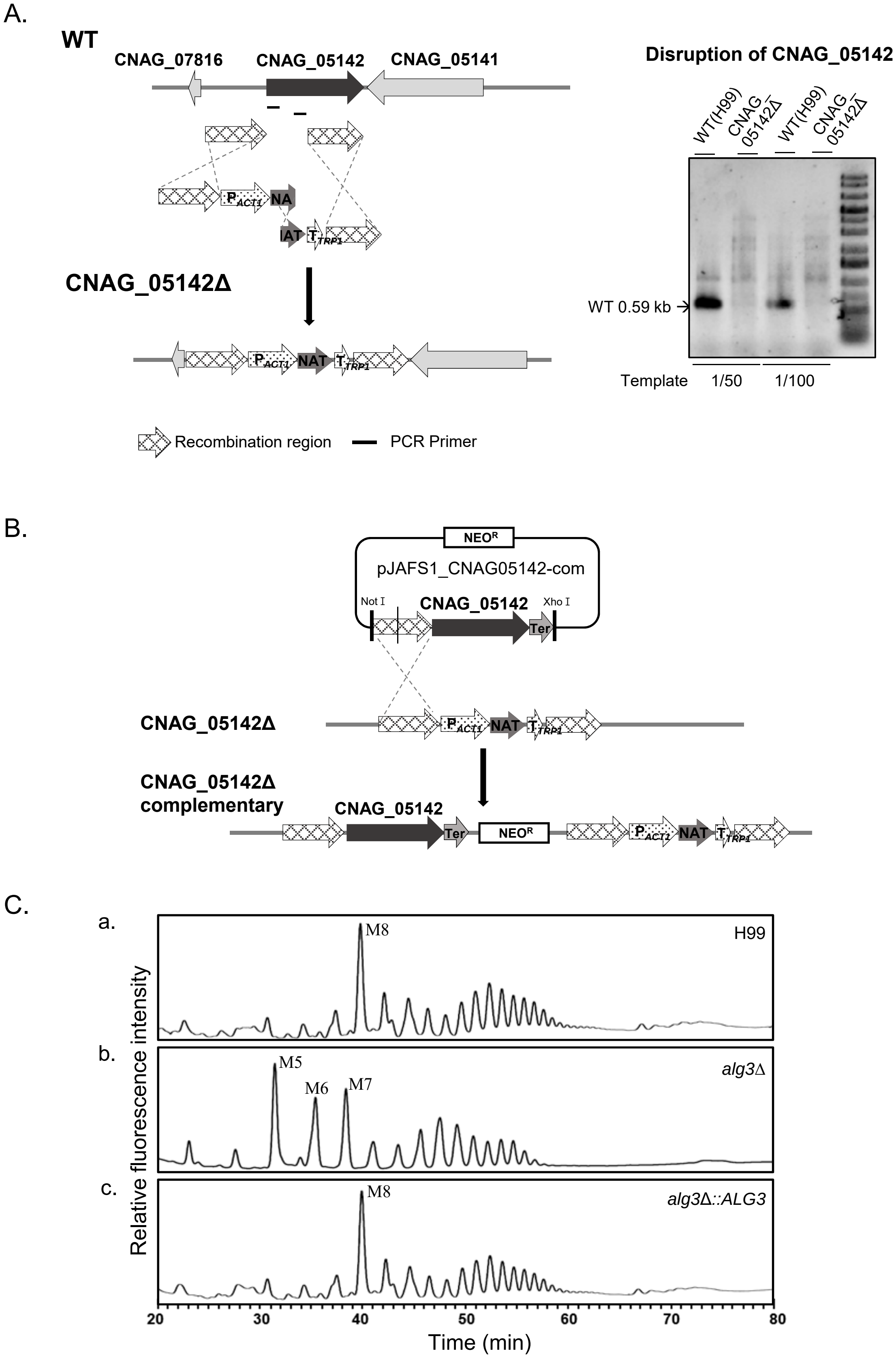

FIG S2 Disruption of *ALG3* and construction of a complementation strain. (A) Strategy for CNAG_05142 disruption using the nourseothricin acetyltransferase (NAT) split marker. Stable transformants were selected on YPD medium containing nourseothricin (100 μg/ml) and were screened by PCR. (B) Scheme of construction of an *alg3*Δ*::ALG3* complementation strain. (C) HPLC-based neutral glycan profiles of WT, *alg3*Δ, and *alg3*Δ*::ALG3* strains.
