# Supplementary material for: Core N-Glycan Structures Are Critical for the Pathogenicity of Cryptococcus neoformans by Modulating Host Cell Death
Source: mBio. 2020 May 12;11(3):e00711-20. doi: 10.1128/mBio.00711-20 (PMC7218283; doi:10.1128/mBio.00711-20)
Supplement: FIG S3 [file mBio.00711-20-sf003.pdf]

Supplementary Fig. 3

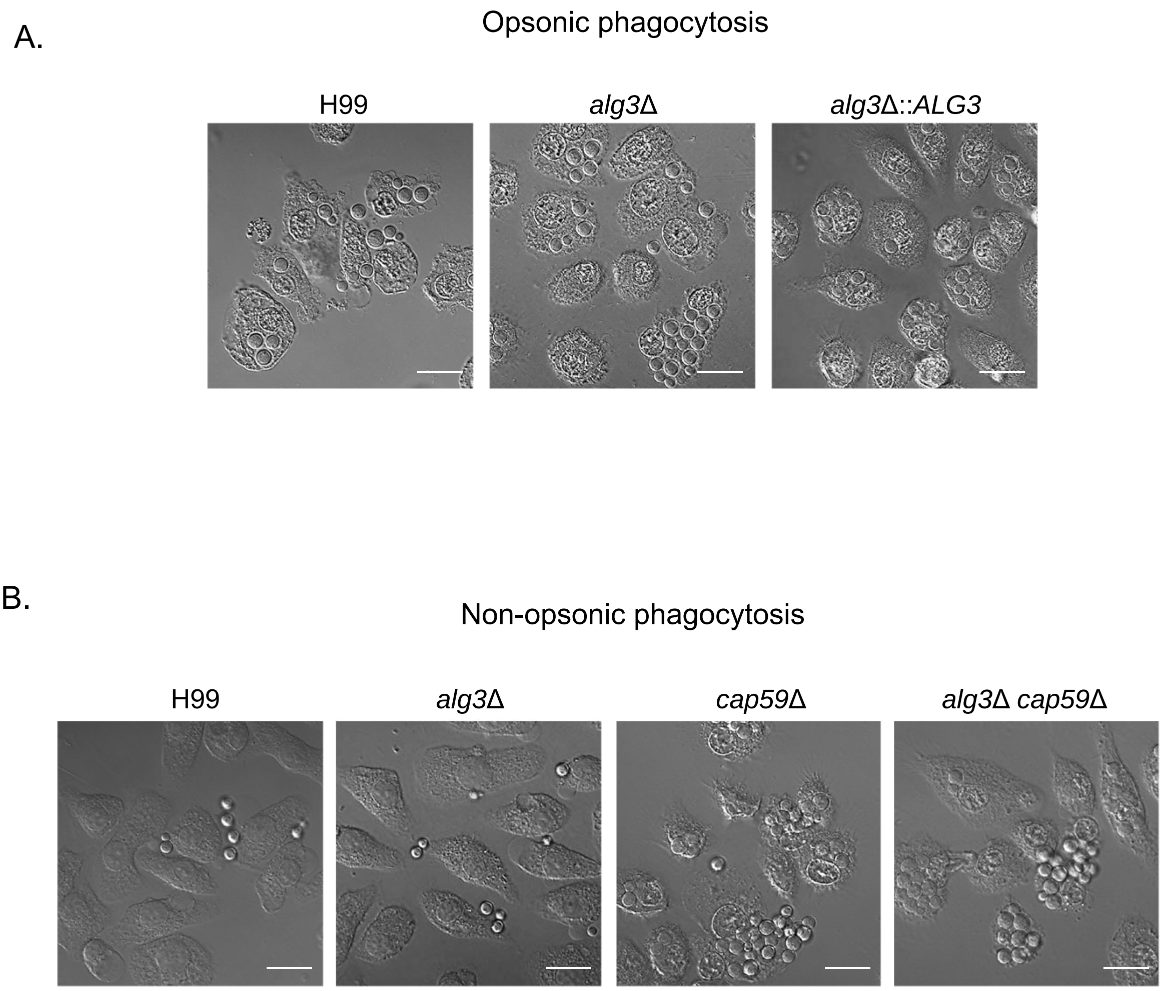

FIG S3 Opsonic and non-opsonic phagocytosis. (A) Encapsular *C. neoformans* cells of WT, *alg3*Δ, and *alg3*Δ::*ALG3* were opsonized with 18B7 antibody and incubated with J774A.1 cells (*C. neoformans*/J774A.1 ratio, 10:1) for 2 h. Then, non-phagocytized yeast cells were removed by washing, and phagocytized yeast cells were evaluated by microscopy. (B) Acapsular yeast cells of WT, *alg3*Δ, *cap59*Δ, and *alg3*Δ *cap59*Δ were incubated with macrophages (*C. neoformans*/J774A.1 ratio, 10:1) for 2 h. Non-phagocytized yeast cells were removed and phagocytized yeast cells were evaluated under a Zeiss confocal microscope. Scale bars, 20 µm.
