# Supplementary figures and images for: Core N-Glycan Structures Are Critical for the Pathogenicity of Cryptococcus neoformans by Modulating Host Cell Death
Source: mBio. 2020 May 12;11(3):e00711-20. doi: 10.1128/mBio.00711-20 (PMC7218283; doi:10.1128/mBio.00711-20)

# Supplementary Fig. 4

A.

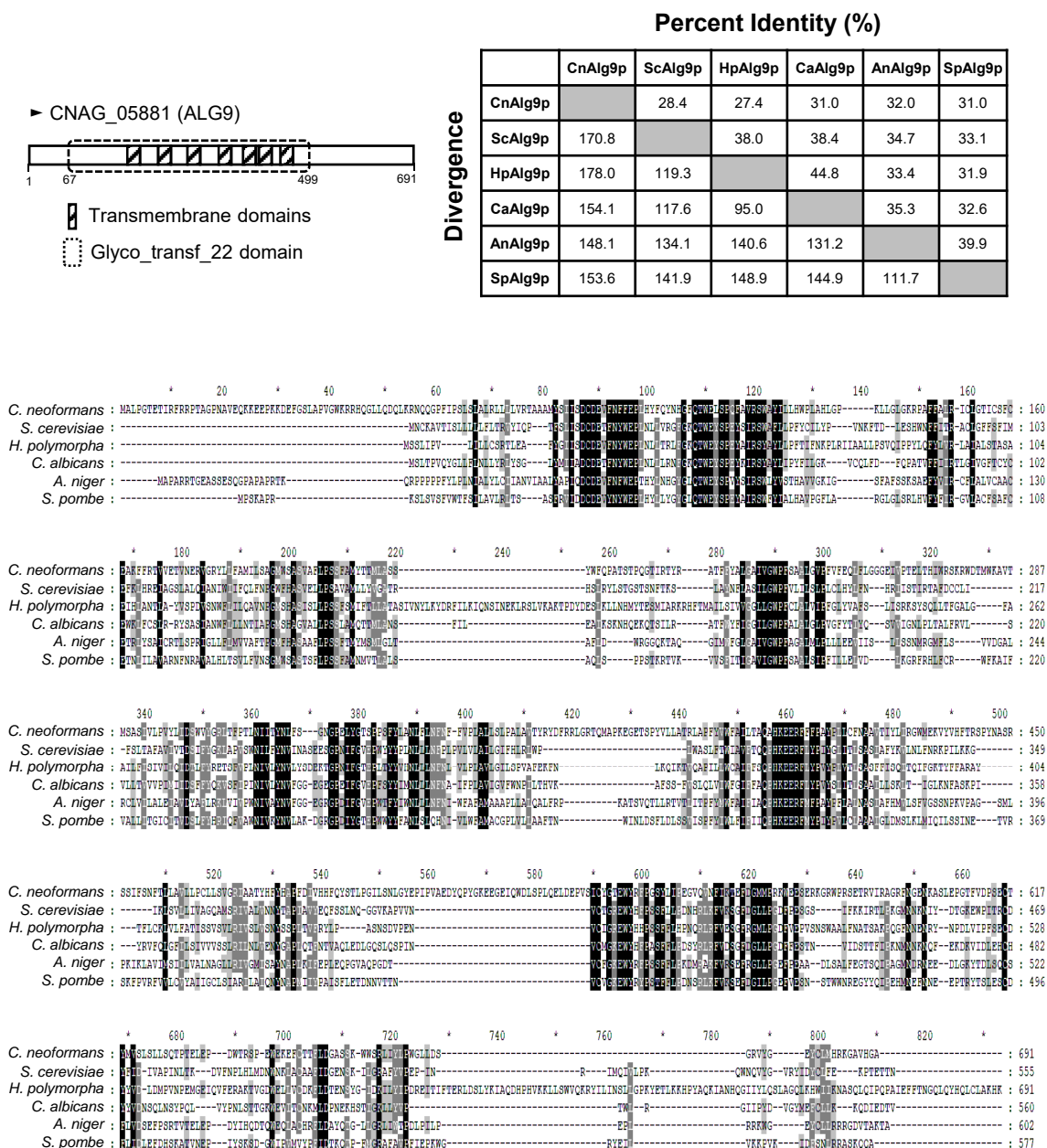

# Supplementary Fig. 4

B.

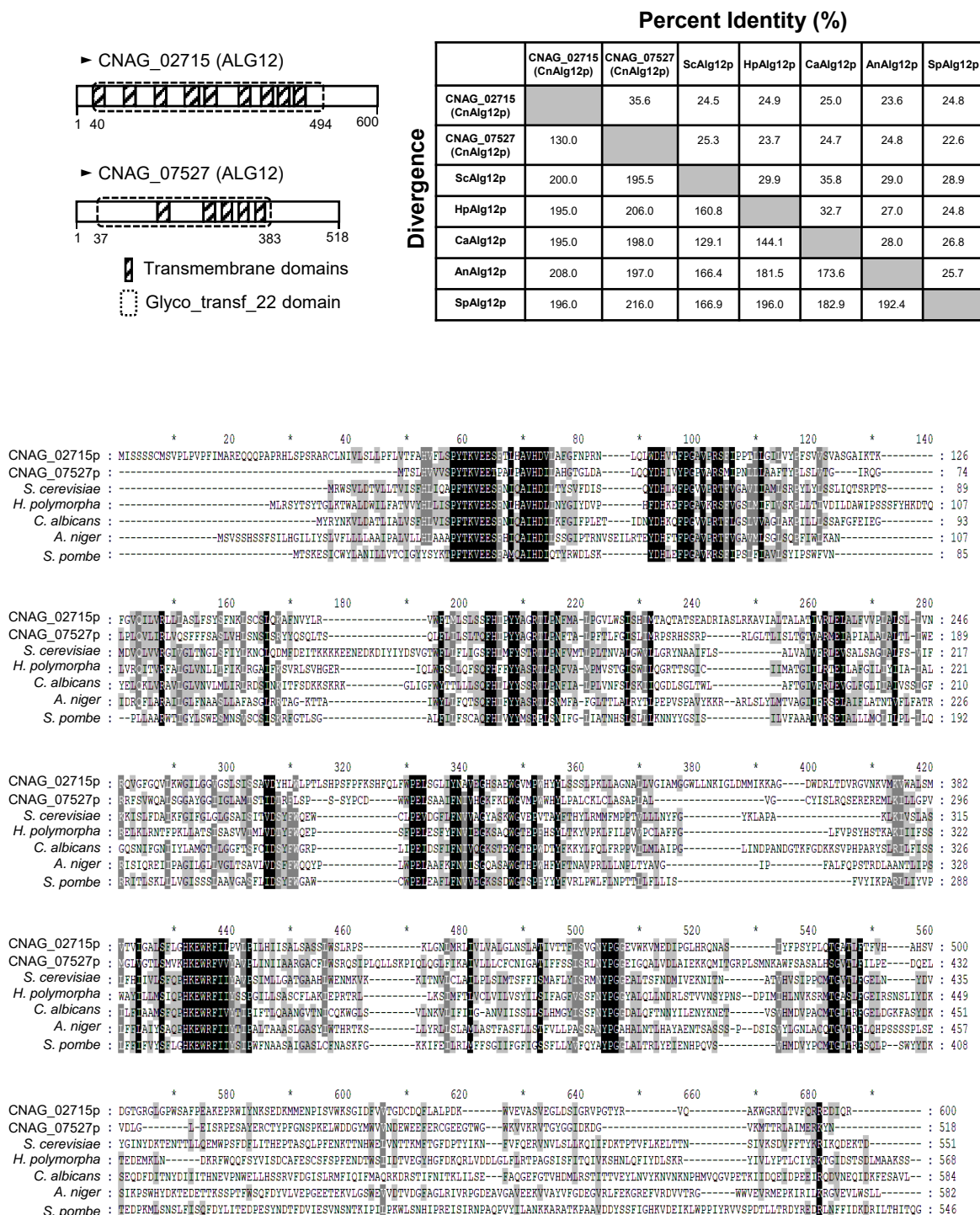

Supplement: FIG S4 [file mBio.00711-20-sf004.pdf]
