# Supplementary material for: Core N-Glycan Structures Are Critical for the Pathogenicity of Cryptococcus neoformans by Modulating Host Cell Death
Source: mBio. 2020 May 12;11(3):e00711-20. doi: 10.1128/mBio.00711-20 (PMC7218283; doi:10.1128/mBio.00711-20)
Supplement: FIG S5 [file mBio.00711-20-sf005.pdf]

Supplementary Fig. 5

A.

B.

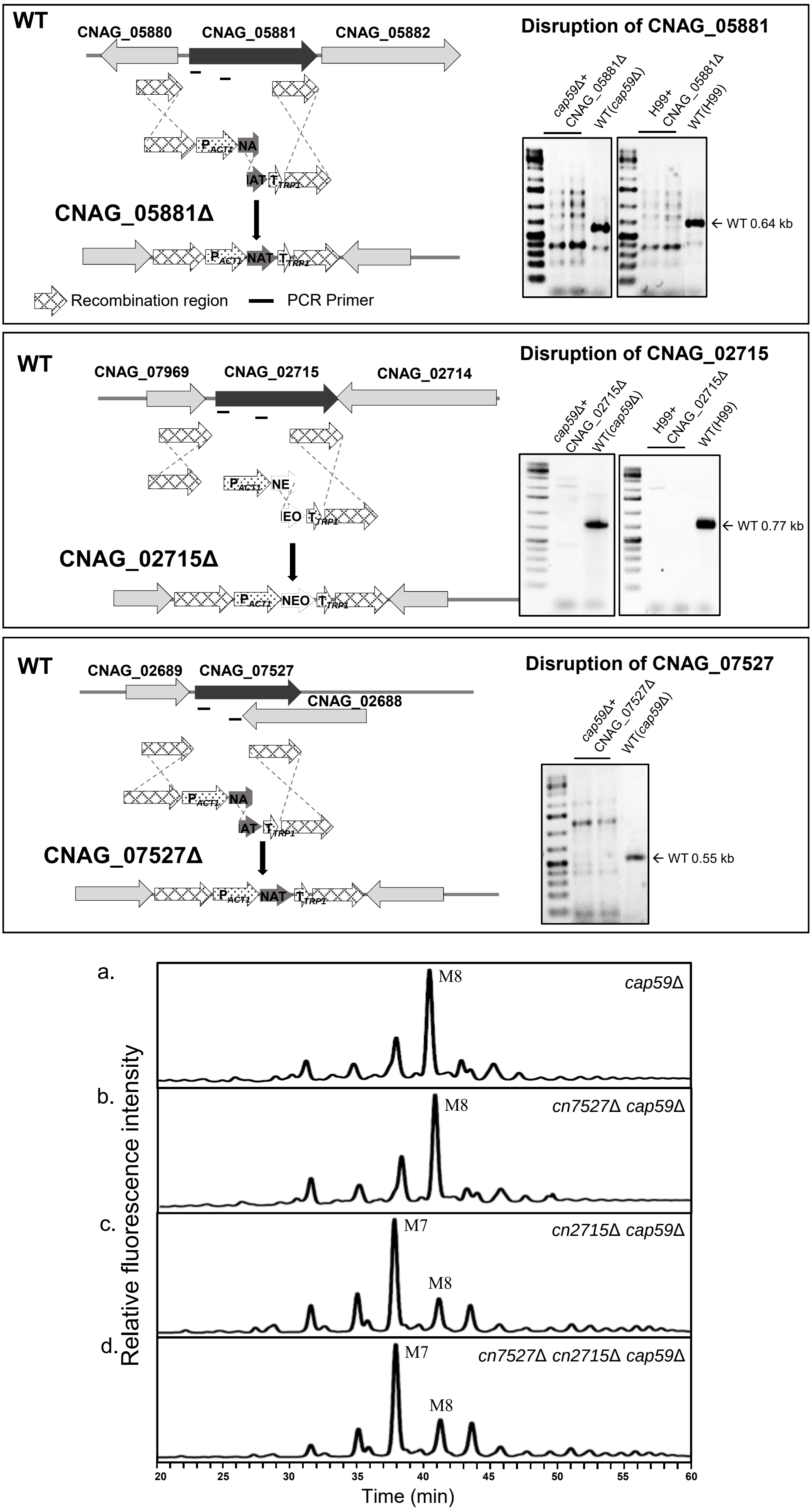

FIG S5 Disruption of *ALG9* and *ALG12*. (A) Strategy for CNAG_05881 and CNAG_07527 disruption using the NAT split marker and CNAG_02715 disruption using the neomycin (*NEO*) split marker. Stable transformants were selected on YPD medium containing nourseothricin (100 μg/ml) and neomycin (200 μg/ml), respectively, and were screened by PCR. (B) HPLC-based neutral glycan profiles of *cap59*Δ, *alg9*Δ *cap59*Δ, and *alg12*Δ *cap59*Δ strains.
