# Supplementary material for: Core N-Glycan Structures Are Critical for the Pathogenicity of Cryptococcus neoformans by Modulating Host Cell Death
Source: mBio. 2020 May 12;11(3):e00711-20. doi: 10.1128/mBio.00711-20 (PMC7218283; doi:10.1128/mBio.00711-20)
Supplement: FIG S6 [file mBio.00711-20-sf006.pdf]

Supplementary Fig. 6

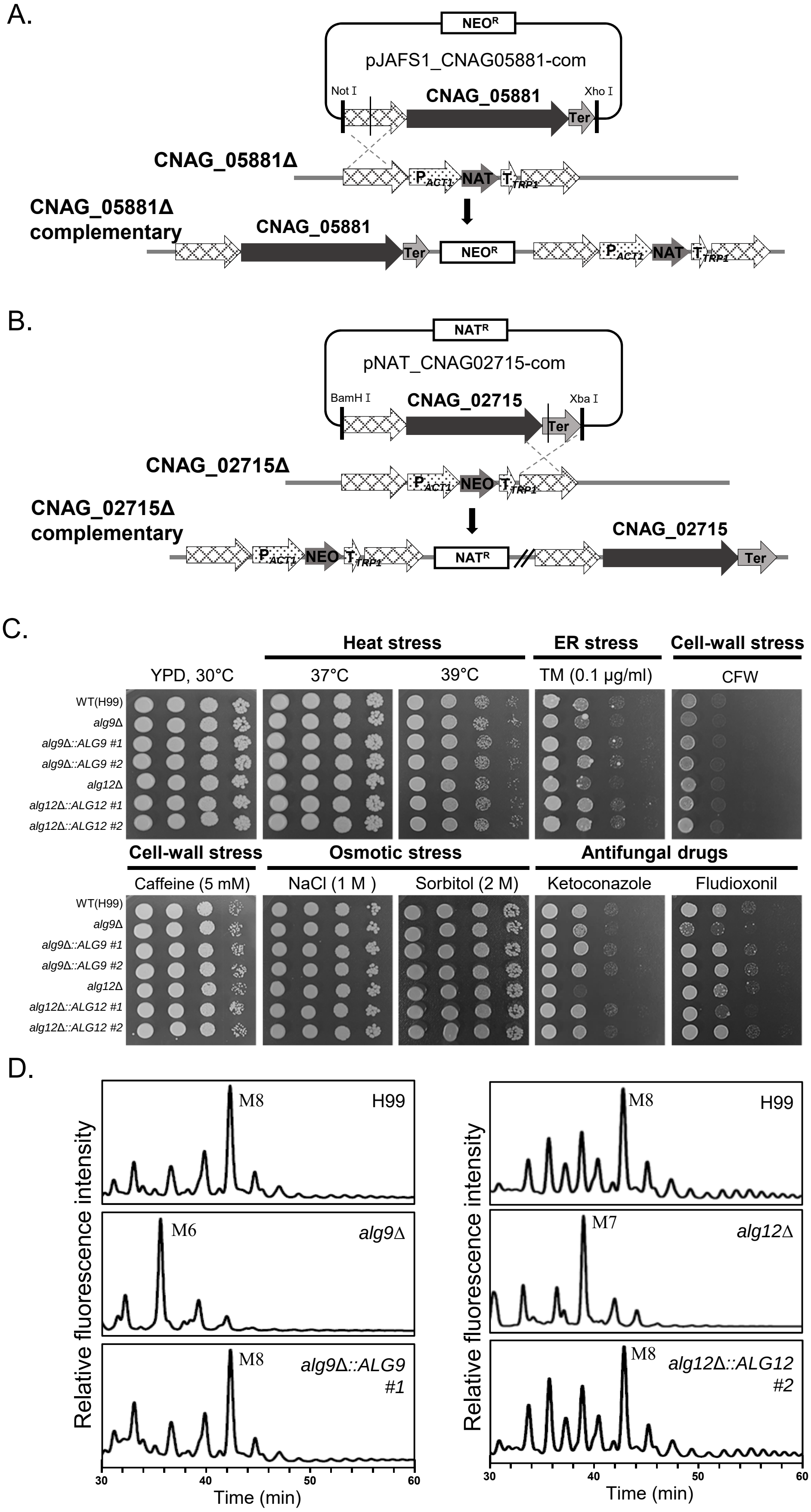

FIG S6 Construction of *ALG9* and *ALG12* complementation strain. (A) Strategy for CNAG_05881 complementary using the *NEO* split marker. Stable transformants were selected on YPD medium containing *NEO*. (B) Strategy for CNAG_02715 complementary using the NAT split marker. Stable transformants were selected on YPD medium containing NAT. (C) Spotting analysis of growth recovery of *C. neoformans alg9*Δ*::ALG9* and *alg12*Δ*::ALG12* strains under heat stress, ER stress, cell-wall stress, osmotic stress, and antifungal drug treatments. (D) HPLC-based neutral glycan profiles of H99, *alg9*Δ, *alg9*Δ::*ALG9* #1, *alg12*Δ and *alg12*Δ::*ALG12* #2 strains.
