# Supplementary material for: Core N-Glycan Structures Are Critical for the Pathogenicity of Cryptococcus neoformans by Modulating Host Cell Death
Source: mBio. 2020 May 12;11(3):e00711-20. doi: 10.1128/mBio.00711-20 (PMC7218283; doi:10.1128/mBio.00711-20)
Supplement: FIG S7 [file mBio.00711-20-sf007.pdf]

Supplementary Fig. 7

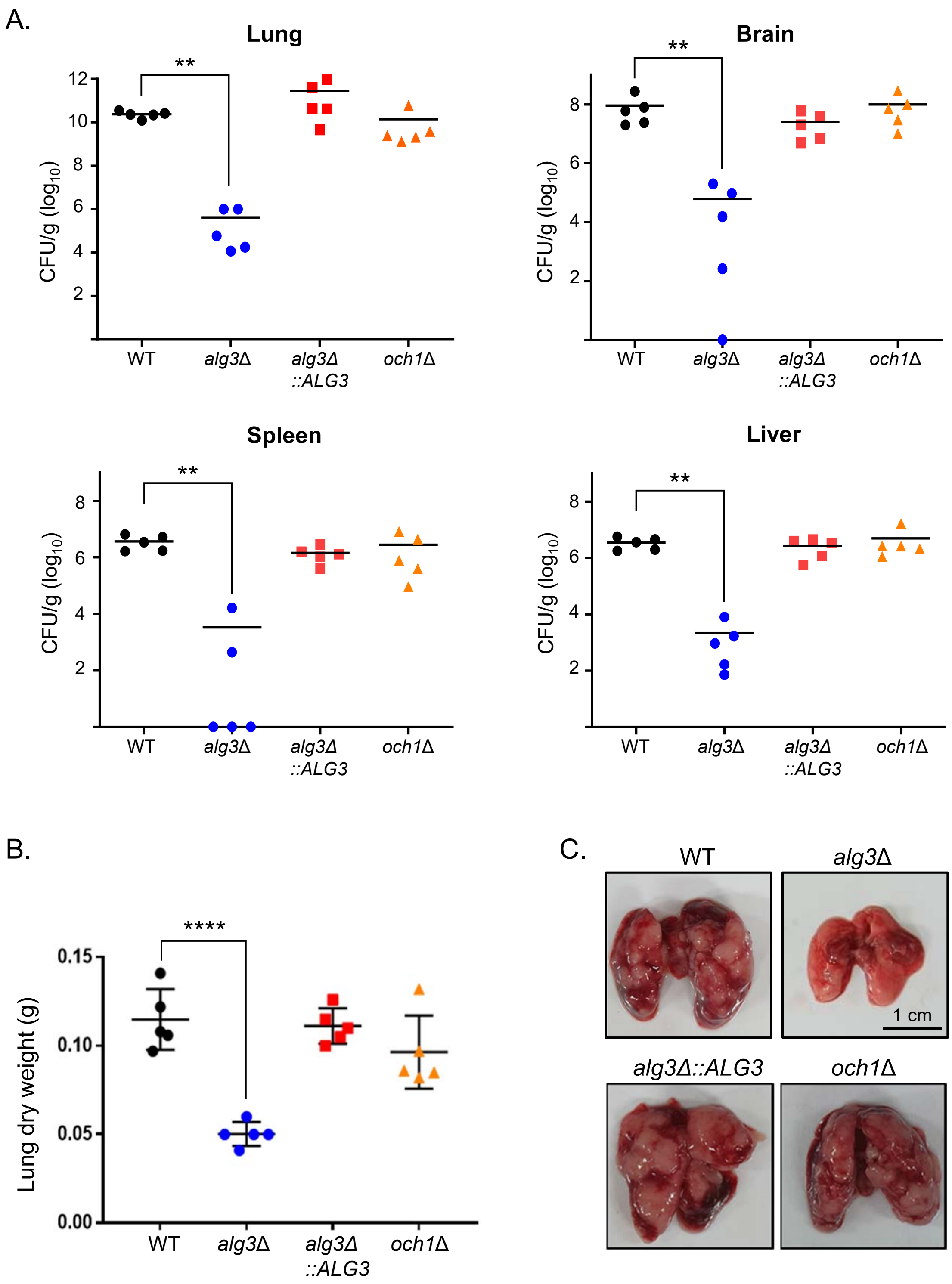

FIG S7 Fungal burden analysis in different organs and lung morphology in the mice infected with *C. neoformans*. (A) Distribution of fungal cells in the systemic organs (lungs, brain, liver and spleen) of infected mice at the humane endpoint of the experiment carried out in Fig.10C. Organs infected with the WT, *alg3*Δ, *alg3*Δ::*ALG3* and *och1*Δ strains were collected and fungal burdens were monitored in organs by determining CFU upon plating on YPD medium. Five mice for each strain were used for the experiments, and horizontal bars in each graph represent the average CFU. (B) Lung dry weight of infected mice. All the statistical analysis was performed using an unpaired two tailed Student's t test (** $P < 0.0005$, ****$P < 0.0001$ WT vs. *alg3*Δ). (C) Representative images of severe swelling and lung injury in infected mice.
