# Supplementary material for: Core N-Glycan Structures Are Critical for the Pathogenicity of Cryptococcus neoformans by Modulating Host Cell Death
Source: mBio. 2020 May 12;11(3):e00711-20. doi: 10.1128/mBio.00711-20 (PMC7218283; doi:10.1128/mBio.00711-20)
Supplement: TEXT S1 [file mBio.00711-20-s0001.docx]

**Text S1. Supplemental Methods and References**

**Construction of *CAP59* deletion cassette and acapsular *C. neoformans* strain *cap59*Δ**

Target genes were disrupted by double-joint PCR, as described previously (1). For the construction of the mutant strain with *CAP59* deletion (*cap59*Δ), DNA fragments containing the 5′- and 3′-flanking regions of *CAP59* (CNAG_00721) were amplified using the primer pairs NheI_CnCAP59D_L1/CnCAP59D_L2_M13F and M13R_CnCAP59D_R1/CnCAP59D_R2_NheI, respectively, and H99 genomic DNA as a template. The 5′- and 3′-regions of HYG-split markers were amplified using the primer pairs HYG_M13F/HYG_L_NheI and HYG_M13R/NheI_HYG_R, respectively, and the plasmid pJAF15 containing hygromycin B marker as a template. *CAP59-HYG* fusion PCR products of 5′- and 3′-flanking regions were generated by overlap PCR using the primer sets NheI_CnCAP59D_L1/HYG_L_NheI and CnCAP59D_R2_NheI/NheI_HYG_R, respectively. The 5′- and 3′-fragments of the *CAP59* disruption cassette were cloned into the T-Blunt^TM^ (SolGent) vector, generating pT-CnCAP59D_L and pT-CnCAP59D_R, respectively. The 5′- and 3′- regions of the *CAP59* disruption cassette were amplified using the primer pairs CnCAP59D_L1/HYG_L and CnCAP59D_R2/HYG_R, respectively, and pT-CnCAP59D_L and pTCnCAP59D_R, respectively, as a template. The *CAP59* disruption cassettes were biolistically transformed into strains H99 and *och1*Δ, respectively, generating *cap59*Δ and *och1*Δ *cap59*Δ (Table S1A). Transformants isolated on YPD_HYG_ were screened by PCR for correct gene disruption.

**Construction of *C. neoformans* *alg3*Δ and *ALG3* complementation strains**

For the construction of the *ALG3* (CNAG_05142) deletion mutant strain, DNA fragments containing the 5′- or 3′-flanking regions of CNAG_05142 ORF were PCR-amplified from H99 genomic DNA with the primer sets CN_05142D_L1/CN_05142D_L2 and CN_05142D_R1/CN_05142D_R2 (Table S1C). The 5′- and 3′-regions of the selectable marker nourseothricin acetyltransferase (NAT) were amplified with the primer sets M13Fe/NSL-2 and M13Re/NSR-2 using pNAT-STM as a template. *ALG3-NAT* fusion products of 5′- and 3′-flanking regions were generated by overlap PCR using the primer sets M13Fe/CN_05142D_L2 and CN_05142D_R1/NSR-2, respectively. The 5′- and 3′-fragments of the *ALG3* disruption cassette were introduced into *C. neoformans* serotype A strain H99 (MATα) by biolistic transformation. Transformants were selected on YPD_NAT_, and gene disruption was screened by PCR (Fig. S2A). To generate complemented strains, a DNA fragment containing *ALG3* was amplified by PCR and subcloned into pJAFS1 containing the G418 resistance marker. The resultant vector was excised at the single *Bst*Z17I site and reintegrated into the native *ALG3* locus of the *alg3*Δ strain by biolistic transformation (Fig. S2B). The complemented strains were confirmed by HPLC for *N*-glycan profiles (Fig. S2C). To generate the *alg3*Δ mutant and the *ALG3*-complemented *alg3*Δ mutant strains in *cap59*Δ background, the 5′- and 3′-regions of the *CAP59* disruption cassette were amplified by PCR and introduced into the *alg3*Δ and *alg3*Δ::*ALG3* strains (Table S1A).

**Construction of *C. neoformans* *alg9*Δ, *alg12*Δ mutants and complementation strains**

For the construction of the *ALG9* (CNAG_05881) deletion mutant strain, DNA fragments containing the 5′- or 3′-flanking region of the CNAG_05881 ORF were amplified by PCR from H99 genomic DNA with the primer sets CN_05881D_L1/CN_05881D_L2 and CN_05881D_R1/CN_05881D_R2 (Table S1C). The 5′- and 3′-flanking regions of the selectable marker NAT were amplified with the primer sets M13Fe/NSL-2 and M13Re/NSR-2, respectively, using pNAT-STM as a template. The *ALG9-NAT* fusion products of 5′- and 3′-flanking regions were generated by overlap PCR using the primer sets M13Fe/CN_05881D_L2 and CN_05881D_R1/NSR-2, respectively. The 5′- and 3′-fragments of the *ALG9* disruption cassette were cloned into the T-Blunt^TM^ vector, generating pT-CnALG9D_L and pT-CnALG9D_R. The 5′- and 3′-regions of the *ALG9* disruption cassette were amplified by PCR and biolistically transformed into strains H99 and *cap59*Δ, respectively. For the construction of the *ALG12* (CNAG_02715) deletion mutant strain, the 5′-flanking or 3′-flanking regions of CNAG_02715 ORF were amplified by PCR from H99 genomic DNA using the primer sets CN_02715D_L1/CN_02715D_L2 and CN_02715D_R1/CN_02715D_R2, respectively (Table S1C). The 5′- and 3′-regions of the selectable marker NEO were amplified with the primer sets M13Fe/B1886 and M13Re/B1887, respectively, using pJAFS1 as a template. The fusion products of 5′-flanking and 3′-flanking regions were generated by overlap PCR using the primer sets M13Fe/CN_02715D_L2 and CN_02715D_R1/NSR-2, respectively. The 5′- and 3′-fragments of the *ALG12-NEO* disruption cassette were cloned into the T-Blunt^TM^ vector, generating pT-CnALG12D_L and pT-CnALG12D_R. The 5′- and 3′-regions of the *ALG12* disruption cassette were amplified by PCR and biolistically transformed into strains H99 and *cap59*Δ. The deletion mutant of CNAG_07527 (cn7527Δ) was constructed using NAT split marker/double-joint-PCR strategies by the same procedure. For the construction of the CNAG_02715/CNAG_07527 double mutant strain, the 5′- and 3′-regions of the CNAG_02715 disruption cassette were PCR-amplified, respectively, and biolistically transformed into the cn7527Δ *cap59*Δ strain (Fig. S5A).

For construction of *ALG9* complemented strains, a DNA fragment containing *ALG9* was amplified by PCR and subcloned into pJAFS1 containing the G418 resistance marker. The resultant vector was excised at the single *BspE*I site located in promoter region and reintegrated into the native *ALG9* locus of the *alg9*Δ strain by biolistic transformation (Fig. S6A). To generate *ALG12* complemented strains, a DNA fragment containing *ALG12* was amplified by PCR and subcloned into pNAT containing the NAT resistance marker. The resultant vector was excised at the single *BsrG*I site located in terminator region and reintegrated into the native *ALG12* locus of the *alg12*Δ strain by biolistic transformation (Fig. S6B). The complemented strains *alg9*Δ*::ALG9* #1, #2 and *alg12*Δ*::ALG12* #1, #2 were tested for growth recovery under various stress conditions (Fig. S6C). The *alg9*Δ*::ALG9* #1 and *alg12*Δ*::ALG12* #2 strains showed growth recovery were used for *N*-glycan profiles by HPLC (Fig. S6D).

**Construction of *C. neoformans* strains expressing his-tagged Plb1 and MP98**

To construct expression vectors for 6XHis-tagged Plb1 and MP98, DNA fragments containing the ORFs of *PLB1* and *MP98* were PCR-amplified with primers containing the 6XHis codon for C-terminal histidine tagging without GPI-anchor (Table S1C). Truncated *PLB1* and *MP98* ORF PCR products were digested with *Kpn*I/*Sma*I and ligated into *Kpn*I/*Sma*I-digested pJAFS1_CNAG_06085Ter and pJAFS1_CNAG_01230Ter, resulting in pJAFS1_CNAG_06085ORF(6HIS) and pJAFS1_CNAG_01230ORF(6HIS) with the G418 resistance marker, respectively. To generate *PLB1* and *MP98* expression strains, the vectors were linearized with *Bgl*II and *Sal*I, respectively, for targeted integration into the native locus via single homologous recombination.

**Supplemental References**

1. Kim MS, Kim SY, Yoon JK, Lee YW, Bahn YS. 2009. An efficient gene-disruption method

in *Cryptococcus neoformans* by double-joint PCR with NAT-split markers. Biochem Biophys Res Commun 390:983-8.

2. Perfect JR, Ketabchi N, Cox GM, Ingram CW, Beiser CL. 1993. Karyotyping of

*Cryptococcus neoformans* as an epidemiological tool. J Clin Microbiol 31:3305-9.

3. Park JN, Lee DJ, Kwon O, Oh DB, Bahn YS, Kang HA. 2012. Unraveling unique structure

and biosynthesis pathway of *N*-linked glycans in human fungal pathogen *Cryptococcus neoformans* by glycomics analysis. J Biol Chem 287:19501-15.

4. Bahn YS, Hicks JK, Giles SS, Cox GM, Heitman J. 2004. Adenylyl cyclase-associated

protein Aca1 regulates virulence and differentiation of *Cryptococcus neoformans* via the cyclic AMP-protein kinase A cascade. Eukaryot Cell 3:1476-91.

5. Esher SK, Ost KS, Kohlbrenner MA, Pianalto KM, Telzrow CL, Campuzano A,  [Nichols CB](https://www.ncbi.nlm.nih.gov/pubmed/?term=Nichols%20CB%5BAuthor%5D&cauthor=true&cauthor_uid=29864141), [Munro C](https://www.ncbi.nlm.nih.gov/pubmed/?term=Munro%20C%5BAuthor%5D&cauthor=true&cauthor_uid=29864141),[Wormley FL Jr](https://www.ncbi.nlm.nih.gov/pubmed/?term=Wormley%20FL%20Jr%5BAuthor%5D&cauthor=true&cauthor_uid=29864141), [Alspaugh JA](https://www.ncbi.nlm.nih.gov/pubmed/?term=Alspaugh%20JA%5BAuthor%5D&cauthor=true&cauthor_uid=29864141). 2018. Defects in intracellular trafficking of fungal cell wall synthases lead to aberrant host immune recognition. PLoS Pathog 14(6): e1007126.

6. Hua J, Meyer JD, Lodge JK. 2000. Development of positive selectable markers for the

fungal pathogen *Cryptococcus neoformans*. Clin Diagn Lab Immunol 7:125-8.

7. Cheon SA, Jung KW, Chen YL, Heitman J, Bahn YS, Kang HA. 2011. Unique evolution of

the UPR pathway with a novel bZIP transcription factor, Hxl1, for controlling pathogenicity of *Cryptococcus neoformans*. PLoS Pathog 7:e1002177.
