# Supplementary material for: Core N-Glycan Structures Are Critical for the Pathogenicity of Cryptococcus neoformans by Modulating Host Cell Death
Source: mBio. 2020 May 12;11(3):e00711-20. doi: 10.1128/mBio.00711-20 (PMC7218283; doi:10.1128/mBio.00711-20)
Supplement: TABLE S1 [file mBio.00711-20-st001.docx]

**Table S1A. Strains used in this study.**

| **Strain** | **Genotype** | **Parent** | **Reference** |
| --- | --- | --- | --- |
| *C*. *neoformans* H99 | *MATα* (serotype A) |  | 2 |
| *alg3*Δ | *MATα* Cn05142::*NAT*#159 | H99 | This study |
| *alg3*Δ::*ALG3* | *MATα* Cn05142::*NAT*#159 Cn05142::*NEO* | *alg3*Δ | This study |
| *alg9*Δ | *MATα* Cn05881::*NAT*#159 | H99 | This study |
| *alg12*Δ | *MATα* Cn02715::*NEO* | H99 | This study |
| *och1*Δ | *MATα* Cn00744::*NAT*#159 | H99 | 3 |
| H99/PLB1(H) | *MATα* Cn06085(H) | H99 | This study |
| *alg3*Δ/PLB1(H) | *MATα* Cn05142::*NAT*#159Cn6085(H) | *alg3*Δ | This study |
| *och1*Δ/PLB1(H) | *MATα* Cn00744::*NAT*#159Cn6085(H) | *och1*Δ | This study |
| *mnn2*Δ/PLB1(H) | *MATα* Cn06872::*NAT*#159Cn6085(H) | *mnn2*Δ | This study |
| H99/MP98(H) | *MATα* Cn01230(H) | H99 | This study |
| *alg3*Δ/MP98(H) | *MATα* Cn05142::*NAT*#159Cn01230(H) | *alg3*Δ | This study |
| *ktr3*Δ/MP98(H) | *MATα* Cn03832::*NAT*#159Cn01230(H) | *ktr3*Δ | This study |
| *hoc3*Δ/MP98(H) | *MATα* Cn00158::*NAT*#159Cn01230(H) | *hoc3*Δ | This study |
| *cac1*Δ | *MATα* *cac1*::*NAT*#159 | H99 | 4 |
| *cap59*Δ | *MATα* Cn00721::*HYB* | H99 | This study |
| *alg3*Δ *cap59*Δ | *MATα* Cn05142::*NAT*#159Cn00721::*HYB* | *alg3*Δ | This study |
| *alg3*Δ::*ALG3cap59*Δ | *MATα*Cn05142::*NAT*#159Cn05142::*NEO* Cn00721::*HYB* | *alg3*Δ::*ALG3* | This study |
| *alg9*Δ *cap59*Δ | *MATα* Cn05881::*NAT*#159Cn00721::*HYB* | *cap59*Δ | This study |
| *alg12*Δ *cap59*Δ | *MATα* Cn02715#159::*NEO*Cn00721::*HYB* | *cap59*Δ | This study |
| *cn7527*Δ *cap59*Δ | *MATα* Cn07527::*NAT#159*Cn00721::*HYB* | *cap59*Δ | This study |
| *cn7527*Δ *cn2715*Δ *cap59*Δ | *MATα* Cn07527::*NAT*Cn00721::*HYB*  *MATα* Cn02715::*NEO* | *cn7527*Δ *cap59*Δ | This study |
| *och1*Δ *cap59*Δ | *MATα* Cn00744::*NAT*#159Cn00721::*HYB* | *och1*Δ | This study |
| *alg9*Δ*::ALG9* | *MATα* Cn05881::*NAT*#159 Cn05881::*NEO* | *alg9*Δ | This study |
| *alg12*Δ*::ALG12* | *MATα* Cn02715::*NEO* Cn02715::*NAT*#159 | *alg12*Δ | This study |
| *mar1*Δ *cap59*Δ | *MATα* *mar1*Δ::*NAT cap59*Δ::*NEO* | *mar1*Δ | 5 |

*Each *NAT-STM#* indicates the Nat^r^ marker with a unique signature tag

**Table S1B. Plasmids used in this study.**

| **Plasmid** | **Description** | **Reference** |
| --- | --- | --- |
| pNAT-STM#159 | NAT-resistant marker vector for gene disruption | 1 |
| pJAF15 | pJAF-based vector containing hygromycin B marker | 6 |
| pJAFS1 | NEO-resistant marker vector | 7 |
| pJAFS1_CNAG_05142Com | pJAFS1 containing the *ALG3* ORF | This study |
| pJAFS1-CNAG_1230His | pJAF-based expression vector for six histidine-tagged MP98 with the G418 (geneticin) marker | This study |
| pJAFS1-CNAG_06085His | pJAF-based expression vector for six histidine-tagged PLB with the G418 marker | This study |
| pT-CnCAP59D_L | pT-Blunt^TM^-based vector containing the CnCAP59D_L fragment | This study |
| pT-CnCAP59D_R | pT-Blunt^TM^-based vector containing the CnCAP59D_R fragment | This study |
| pT-CnALG9D_L | pT-Blunt^TM^-based vector containing the CnALG9D_L fragment | This study |
| pT-CnALG9D_R | pT-Blunt^TM^-based vector containing the CnALG9D_R fragment | This study |
| pT-CnALG12D_L | pT-Blunt^TM^-based vector containing the CnALG12D_L fragment | This study |
| pT-CnALG12D_R | pT-Blunt^TM^-based vector containing the CnALG12D_R fragment | This study |
| pT-CnCN7527D_L | pT-Blunt^TM^-based vector containing the CN7527D_L fragment | This study |
| pT-CnCN7527D_R | pT-Blunt^TM^-based vector containing the CN7527D_R fragment | This study |
| pJAFS1_CNAG_05881Com | pJAFS1 containing the *ALG9* ORF | This study |
| pNAT_CNAG_02715Com | pNAT containing the *ALG12* ORF | This study |

**Table S1C. Oligonucleotides used in this study.**

| **Name** | **Sequence (5′-3′)** | **Purpose** |
| --- | --- | --- |
| CN_05142D_L1 | CTAGCCCATCCTTAACAG | *ALG3* disruption cassette |
| CN_05142D_L2 | GCTCACTGGCCGTCGTTTTACCCAGTAGACTTCCTGAGT | *ALG3* disruption cassette |
| CN_05142D_R1 | CATGGTCATAGCTGTTTCCTGCACTGGGAGTCAAGATGA | *ALG3* disruption cassette |
| CN_05142D_R2 | GAAGTTGTTTCTTATCCATGC | *ALG3* disruption cassette |
| CN_05142Dconfirm_F | CACTGGGAGTCAAGATGA | Confirmation of *ALG3* disruption |
| CN05142Dconfirm_B | GCAGATGACTCACCTAGT | Confirmation of *ALG3* disruption |
| L_Confirm | TCTAGTCGCTGAGAAGGT | Confirmation of NAT marker integration |
| CnD-ACTsqB(B79y) | TGTGGATGCTGGCGGAGGATA | Screening primer for *ACT* promoter |
| M13Fe | GTAAAACGACGGCCAGTGAGC | Screening primer for dominant selectable marker gene (*NAT/NEO*) |
| NSL-2 | AACTCCGTCGCGAGCCCCATCAAC | 5′-Region of *NAT* split marker |
| M13Re | CAGGAAACAGCTATGACCATG | Screening primer for dominant selectable marker gene (*NAT/NEO*) |
| NSR-2 | AAGGTGTTCCCCGACGACGAATCG | 3′-Region of *NAT* split marker |
| Not1alg3comF | AAGCGGCCGCGGACACCAGTTCTTTGTC | *ALG3* complementation primer |
| alg3comXho1B | CGCTCGAGACATTGGTCCTCATGCTG | *ALG3* complementation primer |
| NheI_CnCAP59D_L1 | GCTAGCCAATGTACGTTGCTCTCC | *CAP59* disruption cassette |
| CnCAP59D_L2_M13F | GCTCACTGGCCGTCGTTTTACGTGAGATGCACTTGTCTG | *CAP59* disruption cassette |
| HYG_L_Nhe1 | GCTAGCTCGTCCATCACAGTTTGC | 5′-Region of *HYG* split marker |
| M13R_CnCAP59D_R1 | CATGGTCATAGCTGTTTCCTGGTCCAATCATGCTGGAAC | *CAP59* disruption cassette |
| CnCAP59D_R2_Nhe1 | GCTAGCGGAAAGAAGCAAGGCATG | *CAP59* disruption cassette |
| Nhe1_HYG_R | GCTAGCCGAAGAATCTCGTGCTTTC | 3′-Region of *HYG* split marker |
| CnCAP59D_L1 | CAATGTACGTTGCTCTCC | Amplification of pT-CnCAP59D_L |
| HYG_L | TCGTCCATCACAGTTTGC | Amplification of pT-CnCAP59D_L |
| HYG_R | CGAAGAATCTCGTGCTTTC | Amplification of pT-CnCAP59D_R |
| CnCAP59D_R2 | GGAAAGAAGCAAGGCATG | Amplification of pT-CnCAP59D_R |
| CN_01230O_F_Kpn1 | CTCGGTACCGAGATTGGTCGGCGAAAT | ORF amplification primer |
| CN_01230B_HIS_Sma1 | GACCCGGGTTAGTGGTGGTGGTGGTGGTGACTGCTAGCGTTGTTTGAGC | ORF amplification primer |
| CN_01230T_F_Sma1 | GACCCGGGTGTGATGTGCTTTAGCACG | Terminator amplification primer |
| CN_01230T_B_Not1 | ATGCGGCCGCCAGCAGTAATAGCTCTCC | Terminator amplification primer |
| CN_01230_sq_F | CCTCTTAATCACACAGGC | Confirmation for integration |
| CN_01230_sq_B | CTATGACCATGATTACGC | Confirmation for integration |
| CNAG_06085O_FKpn1 | CTCGGTACCGAGAGAGGTTTGGGTGAT | ORF amplification primer |
| CNAG_06085B_HISSma1 | GACCCGGGTTAGTGGTGGTGGTGGTGGTGACTGGACGCTGTACCAGC | ORF amplification primer |
| CNAG_06085T_FSma1 | GACCCGGGATGGATCGAAGTTATCCTG | Terminator amplification primer |
| CNAG_06085T_BNot1 | ATGCGGCCGCGCTGCCATCAAGTAGCAA | Terminator amplification primer |
| CNAG_06085_sq_F | CTCGGTACCGTTCACCCACTCAGGAAT | Confirmation of integration |
| CNAG_06085_sq_B | CTATGACCATGATTACGC | Confirmation of integration |
| CN_05881D_L1 | GTGTTACTGGATGATAGTGG | *ALG9* disruption primer |
| CN_05881D_L2 | GCTCACTGGCCGTCGTTTTACCAATGAGACAGTTTTGAGTGG | *ALG9* disruption primer |
| CN_05881D_R1 | CATGGTCATAGCTGTTTCCTGGTGGCAAGTTTACCAAGACTC | *ALG9* disruption primer |
| CN_05881D_R2 | GAGATGCAAGAGAAGAATGG | *ALG9* disruption primer |
| CN_05881Dconfirm_F | GTTTGGTAGTCTTGCTCCC | Confirmation of *ALG9* disruption |
| CN_05881Dconfirm_B | CATTCACAGTCTCCACCAC | Confirmation of *ALG9* disruption |
| ALG9_L_Confirm | GATGGCGGAGAAGAAGCAG | Confirmation of NAT marker integration |
| CN_02715D_L1 | GCAACAATCTGGTGCTC | *ALG12* disruption primer |
| CN_02715D_L2 | GCTCACTGGCCGTCGTTTTACCTAATGCGAAGAGAGGAG | *ALG12* disruption primer |
| CN_02715D_R1 | CATGGTCATAGCTGTTTCCTGGGAGATTGGGACAGGTTG | *ALG12* disruption primer |
| CN_02715D_R2 | GCGCGATATTCAACTAGTAG | *ALG12* disruption primer |
| CN_02715Dconfirm_F | CCCACAGGACACAATACTTG | Confirmation of *ALG12* disruption |
| CN_02715Dconfirm_B | CGAGTGACGTTGCTGCTATT | Confirmation of *ALG12* disruption |
| ALG12_L_Confirm_F | CCTTCCCGTCCCTTTCATA | Confirmation of NEO marker integration |
| ALG12_R_Confirm_B | AATTCTGTCCGCCTCGGAA | Confirmation of NEO marker integration |
| CN_07527D_L1 | CTTATTGCCAAAGAGCAGG | *CN7527* disruption primer |
| CN_07527D_L2 | GCTCACTGGCCGTCGTTTTACGCGACTTCTCTGGAAATG | *CN7527* disruption primer |
| CN_07527D_R1 | CATGGTCATAGCTGTTTCCTGCTATCTCCAAGCTCCTATC | *CN7527* disruption primer |
| CN_07527D_R2 | CATATGACCAATTGCCCTC | *CN7527* disruption primer |
| CN_07527Dconfirm_F | CCTCTCTACACGTCGTCG | Confirmation of *CN7527* disruption |
| CN_07527Dconfirm_B | GGTGAGTCTGCGCATCAACAC | Confirmation of *CN7527* disruption |
| CN_07527_L_ Confirm | CCATCGCCCTCATCCTATTCC | Confirmation of NAT marker integration |
| B1886 | TGGAAGAGATGGATGTGC | Primer for 5′ split region of *NEO* |
| B1887 | ATTGTCTGTTGTGCCCAG | Primer for 3′ split region of *NEO* |
| Not1alg9comF | GGGGGCGGCCGCAACCGGTTAAAATGTTGAGTAGA | *ALG9* complementation primer |
| alg9comXho1B | GGGGCTCGAGTGACAGCCTGTCGAGCTCT | *ALG9* complementation primer |
| BamH1alg12comF | GGGGGGATCCAGACTCGATCTTCCAGCAAG | *ALG12* complementation primer |
| alg12comXba1B | GGGGTCTAGAACCTTTTCGGCTCTGACGAT | *ALG12* complementation primer |
